# Opioid Coprescription Through Risk Mitigation Guidance and Opioid Agonist Treatment Receipt

**DOI:** 10.1001/jamanetworkopen.2024.11389

**Published:** 2024-05-15

**Authors:** Jeong Eun Min, Brenda Carolina Guerra-Alejos, Ruyu Yan, Heather Palis, Brittany Barker, Karen Urbanoski, Bernie Pauly, Amanda Slaunwhite, Paxton Bach, Corey Ranger, Ashley Heaslip, Bohdan Nosyk

**Affiliations:** 1 Centre for Advancing Health Outcomes, Vancouver, British Columbia, Canada; 2 BC Centre for Disease Control, Vancouver, British Columbia, Canada; 3 First Nations Health Authority, Vancouver, British Columbia, Canada; 4 School of Public Health and Social Policy, University of Victoria, Victoria, British Columbia, Canada; 5 Faculty of Health Sciences, Simon Fraser University, Burnaby, British Columbia, Canada; 6 Canadian Institute for Substance Use Research, Victoria, British Columbia, Canada; 7 Department of Nursing, University of Victoria, Victoria, British Columbia, Canada; 8 School of Population and Public Health, University of British Columbia, Vancouver, British Columbia, Canada; 9 British Columbia Centre on Substance Use, Vancouver, British Columbia, Canada; 10 Faculty of Medicine, University of British Columbia, Vancouver, British Columbia, Canada; 11 AVI Health and Community Services, Victoria, British Columbia, Canada; 12 Island Medical Program, University of Victoria, Victoria, British Columbia, Canada

## Abstract

**Importance:**

At the onset of the COVID-19 pandemic, the government of British Columbia, Canada, released clinical guidance to support physicians and nurse practitioners in prescribing pharmaceutical alternatives to the toxic drug supply. These alternatives included opioids and other medications under the risk mitigation guidance (RMG), a limited form of prescribed safer supply, designed to reduce the risk of SARS-CoV-2 infection and harms associated with illicit drug use. Many clinicians chose to coprescribe opioid medications under RMG alongside opioid agonist treatment (OAT).

**Objective:**

To examine whether prescription of hydromorphone tablets or sustained-release oral morphine (opioid RMG) and OAT coprescription compared with OAT alone is associated with subsequent OAT receipt.

**Design, Setting, and Participants:**

This population-based, retrospective cohort study was conducted from March 27, 2020, to August 31, 2021, included individuals from 10 linked health administrative databases from British Columbia, Canada. Individuals who were receiving OAT at opioid RMG initiation and individuals who were receiving OAT and eligible but unexposed to opioid RMG were propensity score matched at opioid RMG initiation on sociodemographic and clinical variables. Data were analyzed between January 2023 and February 2024.

**Exposure:**

Opioid RMG receipt (≥4 days, 1-3 days, or 0 days of opioid RMG dispensed) in a given week.

**Main Outcome and Measures:**

The main outcome was OAT receipt, defined as at least 1 dispensed dose of OAT in the subsequent week. A marginal structural modeling approach was used to control for potential time-varying confounding.

**Results:**

A total of 4636 individuals (2955 [64%] male; median age, 38 [31-47] years after matching) were receiving OAT at the time of first opioid RMG dispensation (2281 receiving ongoing OAT and 2352 initiating RMG and OAT concurrently). Opioid RMG receipt of 1 to 3 days in a given week increased the probability of OAT receipt by 27% in the subsequent week (adjusted risk ratio, 1.27; 95% CI, 1.25-1.30), whereas receipt of opioid RMG for 4 days or more resulted in a 46% increase in the probability of OAT receipt in the subsequent week (adjusted risk ratio, 1.46; 95% CI, 1.43-1.49) compared with those not receiving opioid RMG. The biological gradient was robust to different exposure classifications, and the association was stronger among those initiating opioid RMG and OAT concurrently.

**Conclusions and Relevance:**

This cohort study, which acknowledged the intermittent use of both medications, demonstrated that individuals who were coprescribed opioid RMG had higher adjusted probability of continued OAT receipt or reengagement compared with those not receiving opioid RMG.

## Key Points

**Question:**

Is coprescription of hydromorphone tablets or sustained-release oral morphine (opioid risk mitigation guidance [RMG]) and opioid agonist treatment (OAT) associated with subsequent OAT receipt?

**Findings:**

This population-based, retrospective cohort study included 4636 individuals who received at least 1 opioid RMG dispensation and were receiving OAT at the time of first opioid RMG dispensation and their matched unexposed group. The study found that receipt of opioid RMG for 4 days or more was associated with a 46% increase in the probability of OAT receipt in the subsequent week compared with those not receiving opioid RMG.

**Meaning:**

These findings require further investigation to determine safety and isolate the hypothesized therapeutic effectiveness of potential combination formulations of OAT.

## Teaser

This cohort study examines the association of coprescription of hydromorphone tablet or sustained-release oral morphine and opioid agonist treatment (OAT) vs OAT alone with the probability of subsequent OAT treatment among people with opioid use disorder in British Columbia, Canada.

## Introduction

At the onset of the COVID-19 pandemic, the province of British Columbia (BC), Canada, implemented the risk mitigation guidance (RMG), which permitted physicians and nurse practitioners in BC to prescribe select opioids, stimulants, benzodiazepines, and alcohol withdrawal management medications to eligible clients.^1^ Having declared a public health emergency on drug overdose death in 2016, the BC government developed and released this interim clinical guidance in acknowledgment of the potential elevated mortality risk during COVID-19 due to disruptions to harm reduction services, an increasingly toxic illicit drug supply, and conflicting public health guidance that promoted physical distancing but discouraged using drugs when alone. The objectives of the RMG were to prevent the spread of COVID-19 and reduce substance-related harms by offering a harm reduction response to the toxic drug supply and were “not intended for treatment of substance use disorders but rather to support individuals with substance use disorders to self-isolate or social distance and avoid risk to themselves or others.”^1^

Given the novelty of the intervention, the dearth of scientific evidence supporting its safety and effectiveness, and the concerns expressed by the medical community regarding safety to patients and the general public, many prescribers were reluctant to participate. Opioid RMG prescriptions reached 7080 individuals in BC between March 27, 2020, and December 31, 2021,^2^ an estimated 7% of the population of people with opioid use disorder (OUD).^3^ Although use of RMG as a precursor to opioid agonist treatment (OAT) or coprescribed alongside OAT was not an explicit goal or requirement,^1^ the guidance indicates that prescribers should determine medication doses and types collaboratively with their clients, considering ongoing substance use patterns and other medical needs. Otherwise, prescribers should offer OAT according to provincial guidelines or consider increasing OAT dosage and provide carries and delivery as needed.^1^

The practice of prescribing opioids under the auspices of RMG alongside OAT provided a serendipitous opportunity to determine the effectiveness of adding a second opioid prescription (hydromorphone tablets primarily) to standard OAT. In BC, buprenorphine-naloxone supplanted methadone as first-line treatment for OUD in 2017, with methadone recommended as second-line treatment if buprenorphine-naloxone was ineffective or had limitations.^4^ Alternative options, including slow-release oral morphine and injectable diacetylmorphine and hydromorphone, are available,^4^ although their uptake has been minimal, partially due to high regulatory and program cost barriers. All forms of OAT are available in both specialized treatment centers and office-based settings.

Importantly, the objective of OAT is to reach a sufficient dosage that eliminates withdrawal symptoms and craving without excessive sedation.^5^ However, dosing guidelines for OAT have remained constant despite the increasing potency of the illicit drug supply due to illicitly manufactured fentanyl and analogues. Fentanyl was first detected in BC in 2012 and is present in more than 82% of all illicit drug poisoning deaths across the province.^6^ These and other structural and contextual factors have contributed to a 12-year decrease in OAT retention in the province.^7^ The clinical guidelines in BC mirror those of other jurisdictions in including a maximum daily dosage for OAT during induction. However, these thresholds are not supported directly by experimental evidence and are derived from populations whose opioid tolerance is likely not representative of current opioid users in BC.^8^

This study forms part of a broader evaluation of the implementation and impacts of RMG in BC (estimated population of 5.2 million in 2021).^9,10^ The full RMG evaluation includes a population-based analysis of the association of prescriptions with all-cause and overdose mortality and acute care visits using administrative health data,^11^ combined with primary data collection (ie, surveys and interviews) with people who use substances,^12^ service prescribers, and health planners to evaluate experiences of facilitators and barriers to implementation and self-reported effect on health and well-being.^9^ Here, we report findings on OAT retention, a secondary outcome identified within the protocol. We capitalize on BC’s extensive linked health administrative data sets to determine the association of hydromorphone tablet or sustained-release oral morphine (opioid RMG) coprescription vs OAT alone with subsequent OAT receipt among people with OUD.

## Methods

### Sources

We used a linked health administrative database that captures all BC residents accessing OAT and eligible to receive opioid RMG. The study population was linked with 10 population-level administrative databases, capturing physician billing records (Medical Services Plan), hospitalizations (Discharge Abstract Database), medication dispensations (PharmaNet), emergency department visits (National Ambulatory Care Reporting System), perinatal services for all provincial births (BC Perinatal Data Registry), dates and cause of death (Vital Statistics death records), the provincial health insurance plan registration (client roster), admission and discharge records of provincial prisons (BC Corrections), illicit drug poisoning deaths (BC Coroners Service), and receipt of housing and income assistance (Ministry of Social Development and Poverty Reduction).^13^ Linkage was achieved via probabilistic matching by the BC Ministry of Health^14^ and delivered to the investigators stripped of identifying information. Linkage across data sets was accomplished at the record level via unique personal health numbers. Providence Health Care Research Institute and the Simon Fraser University Office of Research Ethics determined that this was a quality assurance and improvement study and thus exempt from research ethics board review as per Article 2.5 of the Tri-Council Policy Statement: Ethical Conduct for Research Involving Humans; therefore, no informed consent was required. This cohort study followed the Strengthening the Reporting of Observational Studies in Epidemiology (STROBE) reporting guideline.^15^

### Study Population

The study population included individuals receiving OAT within the first week of opioid RMG initiation from March 27, 2020, to August 26, 2021 (eAppendix 1 in Supplement 1). We constructed matched comparison groups of individuals who were receiving OAT and eligible but unexposed to opioid RMG prescriptions. Matching was executed on the month of initial opioid RMG dispensation receipt.^16^ Dispensations of OAT were identified by unique drug identification numbers (DINs) or product identification numbers (PINs), differentiating them from opioids for indications of pain. We excluded individuals younger than 18 years, those who were incarcerated, those with a missing birth date or sex, and those with a missing provincial health authority of residence, who most likely resided out of province. Indication of race and ethnicity is not available within these data. Data capture spanned from January 1, 1996, to August 31, 2021, while the study period included information from the calendar week of first OAT receipt or March 27, 2020 (the day after the guidance was released), whichever was later. Baseline (time zero) was the date of first opioid RMG dispensation for the exposure group and the corresponding time-matched unexposed group, and everyone in both groups had OAT at time zero. Once matched at time zero, all study variables were updated on a weekly basis, and follow-up concluded at incarceration, death, or the end of the study period (August 31, 2021), whichever occurred first.

### Key Measures

The primary exposure was opioid RMG receipt, defined as 4 days or more, 1 to 3 days, or zero days of hydromorphone tablets or a 12-hour sustained-release oral morphine (a 24-hour sustained-release oral morphine is used as a form of OAT in BC^4^) dispensed in a given week. There were no unique DINs or PINs assigned to identify medications used for the purposes of RMG prescribing. Thus, we developed algorithms to identify opioid RMG recipients by applying restrictions to our case searches using prescription data from PharmaNet, including prescription history, timing (start date and duration), drug type (by DIN or PIN), and a list of keywords from the freeform codes in the directions for use variable.^9^

The primary outcome was OAT receipt, defined as at least 1 dispensed dose of OAT, in the subsequent week. The binary outcome varied over time until the end of follow-up. Discontinuation of OAT was defined as having a gap of 5 days or more for methadone or slow-release morphine, 6 days or more for buprenorphine-naloxone, and 3 days or more for injectable hydromorphone or diacetylmorphine between prescriptions, according to dose reversion guidelines after a disruption in medication refill adherence.^4^ Because OAT dispensations occurring in hospitals are not recorded in the PharmaNet database, we assumed treatment continued during hospitalization.^17^ Both exposure and outcome were updated on a weekly basis (eTables 1-3 in Supplement 1).

We also considered a selection of other baseline and time-varying covariates hypothesized to be associated with the dependent and primary independent variable. Baseline covariates included the following: age; sex; region of residence (rural vs urban); indicator of Vancouver or South/Central Vancouver Island Health Service Delivery Area, where initial implementation was concentrated^2^; and time since OUD diagnosis (<5, 5-9, or ≥10 y). Time-dependent variables, updated on a weekly basis, included indicators of unstable housing, receipt of income assistance, OAT receipt in the last week (no OAT, OAT guideline noncompliant, or OAT guideline compliant based on opioid dosage), comorbidity (mental health conditions, alcohol use disorder, other substance use disorder, HIV, hepatitis C virus, or chronic pain), tobacco use, receipt of cancer or palliative care, Carlson Comorbidity Index, chronic disease score, drug overdose in the last 30 days, incarceration in a provincial corrections facility in the past year, physician attachment (based on general practitioner visits in the past year), receipt of an opioid-based medication for pain, receipt of stimulant RMG (methylphenidate or dextroamphetamine), and receipt of a benzodiazepine, dispensed either under the RMG or for other indications. Diagnostic codes and supporting references used to identify covariates are provided in eTables 4 and 5 in Supplement 1.

### Statistical Analysis

Analyses were conducted between January 2023 and February 2024. We aimed to balance the distribution of observed baseline characteristics of those exposed and not exposed to opioid RMG prescriptions to reduce confounding. To create the propensity score (PS) model, the probability of opioid RMG receipt was modeled as a function of measured covariates at time zero (eAppendix 2 in Supplement 1). We used logistic regression models to estimate PSs, adjusting for investigator-defined covariates described above and with the additional proxy variables identified by the high-dimensional propensity score (hdPS) algorithm. The hdPS is an automated data-driven approach to derive important proxy variables associated with both exposure and outcome from administrative data for inclusion in PS models.^18^ We identified 50 empirical variables from the physician billing, hospitalization, and pharmacy dispensation databases (eTable 6 in Supplement 1).

We matched exposed and unexposed groups with a 1:1 ratio at the calendar month of opioid RMG initiation from March and April 2020 to August 2021 (17 months in total) based on OAT status and their estimated PS (eAppendix 3 in Supplement 1). Covariates for PS models were measured at the first week of RMG receipt in the exposed group and the first week on OAT within the calendar month for their matched controls. Once selected in the unexposed group in an early month, the person could not be selected again in the unexposed group in the later months. Opioid RMG recipients could be selected into the unexposed group before their first RMG dispensation (and then censored once RMG was initiated) (eFigure 1 in Supplement 1). We executed matching without replacement using 2 distinct matching procedures: nearest neighbor matching using the PS estimated with investigator-defined covariates only^19,20^ and nearest neighbor matching using the PS estimated with the additional hdPS covariates. The hdPS covariate selection and matching were performed using a SAS macro, version 2 (SAS Institute Inc).^21^ The distribution of the baseline covariates was assessed by standardized mean differences (SMDs) between the exposed and unexposed groups. For good variable balance, the absolute SMD should be less than or equal to 0.1.^22^

After we constructed the matched cohorts at time zero, we measured crude incident rates of OAT receipts by the number of weeks receiving OAT divided by total person-weeks, stratified by opioid RMG receipt within a given week. Marginal structural models are designed to handle cases in which time-dependent variables are simultaneously confounders of the outcome of interest and are associated with previous treatment.^23^ Past OAT exposure can be considered a time-dependent confounder for the association of opioid RMG with future OAT receipt because it may be hypothesized to be associated with not only future OAT receipt but also subsequent initiation of opioid RMG; otherwise, past opioid RMG receipt is independently associated with subsequent OAT exposure. To control for time-varying confounding, stabilized inverse probability of treatment weights and inverse probability of censoring weights were estimated using multinomial logistic regressions and binomial logistic regressions, respectively, for each time point of the study. Follow-up was censored at incarceration, RMG initiation if selected for the unexposed group, death, or the end of the study period. The final weights were calculated by the product of the estimated inverse probability of treatment weights and inverse probability of censoring weights, truncated at the 1st and 99th percentiles. We estimated the denominators of the weights given baseline variables, the prior 2 weeks of exposure history, and time-varying covariates measured within the prior week. The numerators of the weights, which depend on baseline variables, were estimated for stabilization. We included identical sets of covariates for treatment and censoring weights listed in Table 1 (time-updated covariates noted) and included the number of weeks since time zero (a linear and a quadratic term) and the calendar month of time zero (a linear and a quadratic term). The final weighted structural model was executed using a generalized estimating equation regression model with a log link, Poisson distribution, and an independent working covariate matrix to estimate the risk ratio of OAT receipt in the subsequent week.^24^ The model included a time-varying indicator of opioid RMG receipt at current week and baseline covariates, and robust variance estimates were used to calculate confidence limits. All analyses were executed using SAS software, version 9.4 (SAS Institute Inc) and R, version 4.2.2 (R Foundation for Statistical Computing).

**Table 1.** Baseline Characteristics of Opioid RMG Recipients and Nonrecipients Receiving OAT Matched via High-Dimensional Propensity Score at the Time of First RMG Dispensation

Characteristic	RMG^a^		SMD between RMG and non-RMG recipients	
	No opioid (n = 4633)	Opioid (n = 4633)	Before matching	After matching
Sex				
Female	1654 (35.7)	1678 (36.2)	0.03	0.01
Male	2973 (64.3)	2955 (63.8)	−0.03	−0.01
Age, median (IQR), y	38 (31-47)	38 (31-47)	NA	NA
Age group, y				
18-29	953 (20.6)	911 (19.7)	0.06	−0.01
30-39	1639 (35.4)	1614 (34.8)	0.08	−0.01
40-49	1179 (25.4)	1177 (25.4)	0.01	0.00
≥50	862 (18.6)	931 (20.1)	−0.15	0.01
Rural region	461 (10)	455 (9.8)	−0.01	0.00
Vancouver or South/Central Vancouver Island	2255 (48.7)	2433 (52.5)	0.21	0.04
Receipt of income assistance in the past year	3869 (83.5)	3856 (83.2)	0.25	0.00
Unstable housing in the past year^b^	1711 (36.9)	1753 (37.8)	0.26	0.01
OAT in the last week, none^b^	1799 (38.8)	2053 (44.3)	0.41	0.05
OAT guideline compliance				
Noncompliant	716 (15.5)	492 (10.6)	−0.24	−0.05
Compliant^c^	2118 (45.7)	2088 (45.1)	−0.17	−0.01
Time since OUD diagnosis, y				
<5	2270 (49)	2143 (46.3)	0.09	−0.03
5-9	1012 (21.8)	1079 (23.3)	−0.01	0.01
≥10	1351 (29.2)	1411 (30.5)	−0.08	0.01
Charlson Comorbidity Index >0^b^	242 (5.2)	278 (6)	0.01	0.01
Chronic Disease Score, median (IQR)^b^	1.8 (1.3-2.5)	1.8 (1.3-2.5)	0.09	0.00
Overdose acute care visits in the past 30 d^b^	246 (5.3)	247 (5.3)	0.04	0.00
Substance use disorder (ever)^b^	4094 (88.4)	4174 (90.1)	0.07	0.02
Alcohol use disorder (ever)^b^	1931 (41.7)	2032 (43.9)	0.10	0.02
Severe mental health disease (ever)^b^	1270 (27.4)	1356 (29.3)	0.05	0.02
HIV (ever)^b^	307 (6.6)	339 (7.3)	0.04	0.01
HCV (ever)^b^	1030 (22.2)	1030 (22.2)	0.04	0.00
Chronic pain in the past year^b^	1176 (25.4)	1198 (25.9)	−0.03	0.00
Tobacco use disorder in the past year^b^	673 (14.5)	674 (14.5)	−0.03	0.00
Any cancer or palliative care in the past year^b^	358 (7.7)	354 (7.6)	−0.01	0.00
Incarcerated in the past year^b^	366 (7.9)	403 (8.7)	0.06	0.01
Physician attachment, single GP >50% of physician visits^b^	2691 (58.1)	2396 (51.7)	−0.27	−0.06
Opioid dispensations in the past 60 d^b^	774 (16.7)	852 (18.4)	0.12	0.02
Benzodiazepine dispensations in the past 60 d^b^	223 (4.8)	220 (4.7)	−0.03	0.00
Stimulant dispensations in the past 60 d^b^	177 (3.8)	182 (3.9)	0.01	0.00

Abbreviations, GP, general practitioner; HCV, hepatitis C virus; NA, not applicable; OAT, opioid agonist treatment; OUD, opioid use disorder; RMG, risk mitigation guidance; SMD, standardized mean difference.

^a^ The RMG prescriptions included hydromorphone tablets or sustained-release oral morphine.

^b^ Time-varying covariates used in the treatment and censoring weight models.

^c^ Based on the maximum daily dose per week from the 4th week of OAT episode initiation (≥80 mg for methadone; ≥16 mg/3 mg for buprenorphine/naloxone; ≥600 mg for slow-release oral morphine; ≥200 mg for injectable hydromorphone; ≥400 mg for injectable diacetylmorphine; 100 mg or 300 mg per month for extended-release buprenorphine; ≥80 mg for tablet injectable hydromorphone).

We considered several sensitivity analyses to determine the robustness of our results. First, we calculated E-values to assess the robustness to potential unmeasured confounding.^25^ In addition, we conducted subgroup analyses among individuals initiating OAT and opioid RMG within the same week and those already receiving OAT at opioid RMG initiation to determine whether the impact of opioid RMG receipt differed according to when it was initiated at different stages of OAT engagement. Moreover, individuals were stratified by OAT medication type at time zero to determine whether the added prescription had differential effectiveness among clients receiving buprenorphine-naloxone, methadone, or other forms of OAT, including slow-release oral morphine, injectable hydromorphone or diacetylmorphine. Furthermore, to examine whether the benefits of opioid RMG were concentrated during OAT induction, we truncated observations up to 4 weeks of follow-up among those initiating OAT at time zero.

We also used a marginal structural Cox proportional hazards regression model that included measured baseline covariates at OAT initiation and time-varying covariates (linear and quadratic) for cumulative duration of weekly RMG (having at least 1 day of opioid RMG per week) to compare time to OAT discontinuation between continuous opioid RMG receipt vs no opioid RMG among those initiating OAT at time zero. We reset the cumulative exposure variables to zero if opioid RMG was discontinued, assuming that the effect dissipated after discontinuation. We then used the model to estimate 2 per-protocol standardized survival curves: one for when all individuals received opioid RMG and one for when all did not. The 95% CIs for the risk ratio were estimated using 500 bootstrap samples.^26^

Finally, we considered 3 alternate measures of exposure. The exposure group was defined as 6 days or more, 1 to 5 days, or no opioid RMG per week as an alternate threshold of exposure. To account for variance in daily opioid RMG dosing, we reclassified exposure as opioid RMG dispensation of at least 2000 morphine milligram equivalents (MME), up to 2000 MME, or no opioid RMG in a given week based on the weekly median of opioid RMG dispensations. Lastly, to account for the history of exposure, we added cumulative duration of weekly opioid RMG in past 4 weeks (linear and quadratic terms), in addition to the most recent week, to the model.

## Results

**Figure 1.**
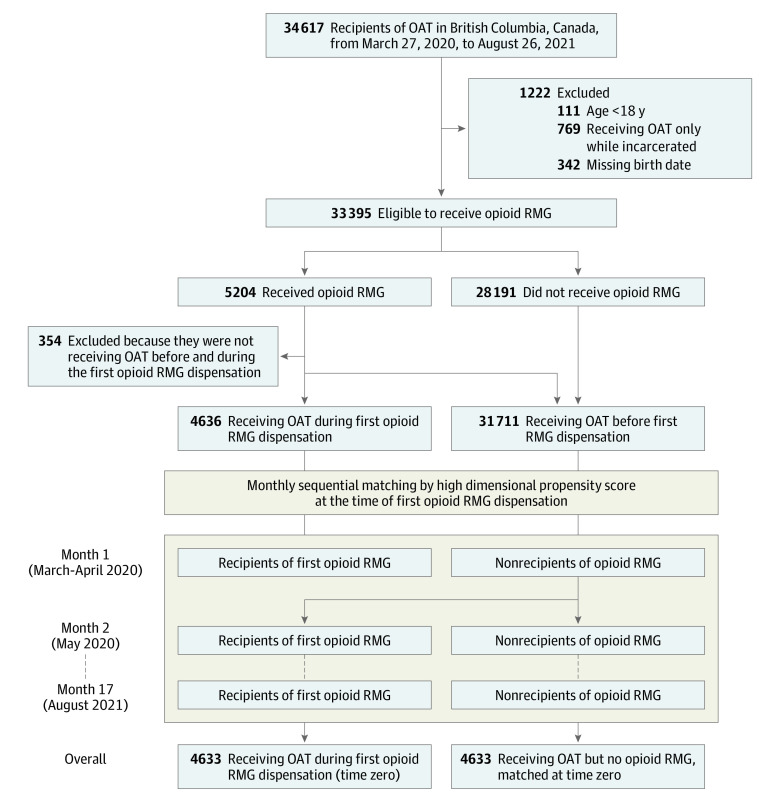
Cohort Construction Time zero is the week of risk mitigation guidance (RMG) initiation. OAT indicates opioid agonist treatment.

A total of 4636 individuals initiated at least 1 opioid RMG dispensation (95% hydromorphone, 8% sustained-release oral morphine, and 2% both) and received OAT in the same week from March 27, 2020, to August 26, 2021 (Table 1). A total of 31 711 individuals were considered as candidates for the unexposed group before matching (Figure 1; eTable 7 in Supplement 1). Before matching, opioid RMG recipients were younger than nonrecipients (SMD for those aged ≥50 years, −0.15), and a higher percentage had not received OAT in the past week (SMD for no OAT, 0.41). A higher percentage of opioid RMG recipients had received income assistance (SMD, 0.25), had an indication of housing instability (SMD, 0.26), and lived in Vancouver or south/central Vancouver Island (SMD, 0.21). Opioid RMG recipients were also less attached to their primary care practitioner (SMD, −0.27) and more likely to have a diagnosed alcohol use disorder (SMD, 0.10). After matching by hdPS, 4633 RMG recipients (2955 [64%] male and 1678 [36%] female; median [IQR] age, 38 [31-47] years) were assessed, and the covariates were balanced between the exposed and unexposed groups, indicated by low (<0.1) absolute SMD (eFigure 2 and eTable 8 in Supplement 1).

**Figure 2.**
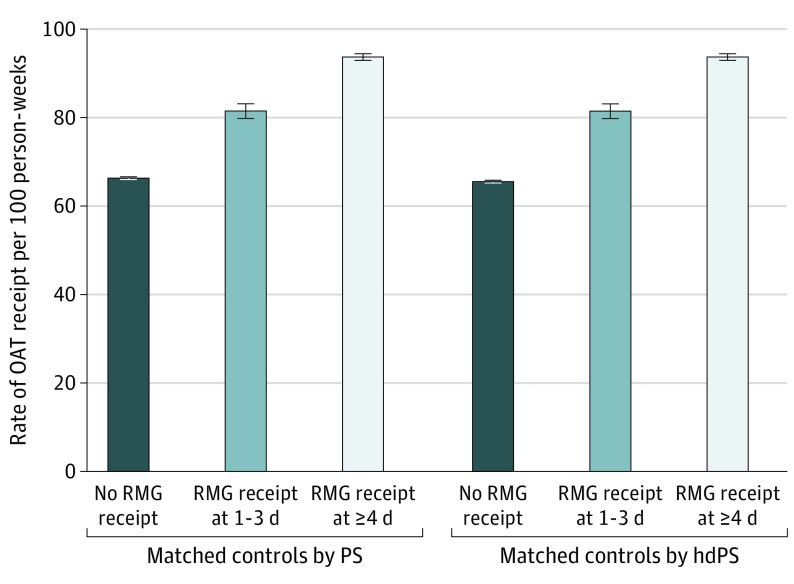
Crude Rates of Opioid Agonist Treatment (OAT) Receipt Among Opioid Risk Mitigation Guidance (RMG) Recipients and Matched Controls Who Were Receiving OAT at Time Zero, March 27, 2020, to August 31, 2021 Time zero is the week of RMG initiation. Whiskers represent 95% CIs. hdPS indicates high-dimensional propensity score; PS, propensity score.

Among individuals receiving OAT at opioid RMG initiation and the matched unexposed group, crude rates of OAT receipt during the study period were higher during periods of receiving opioid RMG compared with not receiving opioid RMG. Those receiving opioid RMG 1 to 3 days per week received OAT in the subsequent week in 81 of 100 person-weeks, whereas those receiving 4 days or more of opioid RMG received OAT in 94 of 100 person-weeks (Figure 2). In contrast, individuals not receiving opioid RMG prescriptions received OAT in the subsequent week in 66 of 100 person-weeks.

After hdPS matching and adjusting for time-varying confounding via marginal structural modeling (see eFigure 3 in Supplement 1 for the distribution of weights), we found that receipt of opioid RMG up to 3 days in a given week increased the probability of OAT receipt in the subsequent week by 27% (adjusted risk ratio [ARR], 1.27; 95% CI, 1.25-1.30), whereas receipt of RMG for 4 days or more increased the probability of receipt in the subsequent week by 46% (ARR, 1.46; 95% CI, 1.43-1.49) compared with those not receiving RMG in the current week (Table 2; eTables 9-10 in Supplement 1). The E-values for the estimates are 1.86 (E-value for lower CI, 1.81) for RMG up to 3 days and 2.28 (E-value for lower CI, 2.21) for RMG of 4 days or more, indicating that substantial unmeasured confounding would be needed to reduce the observed association or its CI to null (eFigure 4 in Supplement 1).

**Table 2.** Association of Opioid RMG Receipt With OAT Receipt Using High-Dimensional Propensity Score–Matched Cohorts

Analysis	No. of individuals^b^	Adjusted risk ratio (95% CI) for RMG vs no RMG^a^	
		RMG 1-3 d	RMG ≥4 d
Primary analysis	4633	1.27 (1.25-1.30)	1.46 (1.43-1.49)
Subgroup analyses^c^			
People initiating OAT and opioid RMG concurrently	2352	1.56 (1.50-1.62)	2.04 (1.97-2.11)
During OAT induction (up to 4 weeks)	2352	1.06 (1.03-1.09)	1.42 (1.38-1.45)
People initiating OAT before opioid RMG initiation	2281	1.07 (1.05-1.09)	1.15 (1.12-1.17)
People initiating or receiving methadone	2859	1.22 (1.19-1.25)	1.40 (1.37-1.43)
People initiating or receiving buprenorphine-naloxone	427	1.34 (1.18-1.53)	1.56 (1.40-1.73)
People initiating or receiving other forms of OAT	1348	1.27 (1.21-1.32)	1.49 (1.44-1.54)

Abbreviations: OAT, opioid agonist treatment; RMG, risk mitigation guidance.

^a^ The RMG prescriptions included hydromorphone tablets or sustained-release oral morphine.

^b^ Number of individuals receiving RMG, matched with unexposed group. The marginal structural model with inverse probability of treatment weights controlled for age; sex; rurality of residence; service access (Vancouver or South/Central Vancouver Island vs others); income assistance receipt in the past 12 months; prior-week OAT dispensation (none, suboptimal, or optimal); unstable housing in the past 2 months; years since the first opioid use disorder indication (<5, 5-9, or ≥10); overdose-related acute care visits in the past 30 days; Charlson Comorbidity Index (>0); Chronic Disease Score; any prior indication of disorder for substance use, alcohol use, mental health, HIV, hepatitis C virus, or chronic pain in the past 12 months; tobacco use in the past 12 months; any cancer or palliative care in the past 12 months; incarcerated in the past 12 months; physician attachment in the past 12 months; opioid dispensation other than OAT prescription in the past 60 days; benzodiazepine dispensation in the past 60 days; week of follow-up (linear and quadratic); and calendar month of time zero from 1 (March to April 2020) to 17 (August 2021) (linear and quadratic).

^c^ On the basis of RMG initiation week for the exposure group and the matched unexposed group.

This association appeared to be strongest among individuals initiating OAT and opioid RMG concurrently; those receiving RMG for 4 days or more were 2 times more likely to receive OAT the following week compared with those not receiving opioid RMG prescriptions (ARR for RMG ≥4 days, 2.04; 95% CI, 1.97-2.11) (Table 2). Nevertheless, there was still an association for those who added RMG prescriptions while already engaged in OAT (ARR for RMG ≥4 days, 1.15; 95% CI, 1.12-1.17).

**Figure 3.**
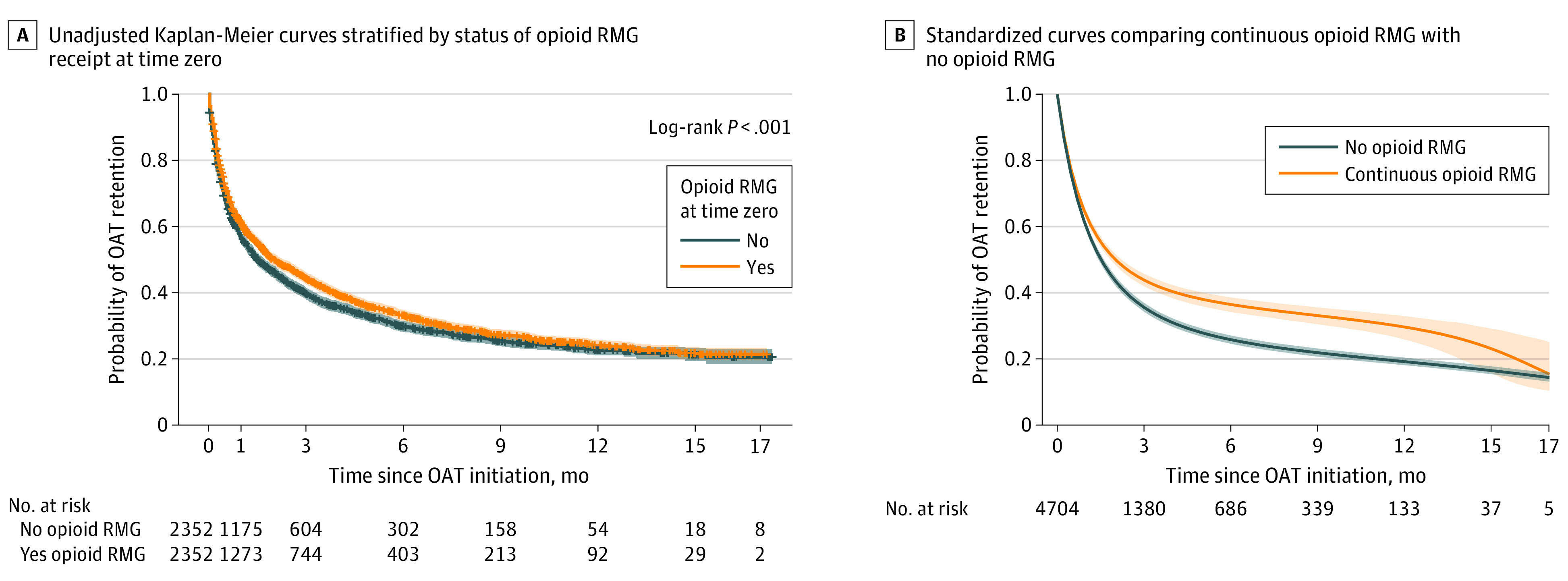
Survival Curves on the Probability of Opioid Agonist Treatment (OAT) Retention Among Opioid Risk Mitigation Guidance (RMG) Recipients and High-Dimensional Propensity Score–Matched Unexposed Group Initiating OAT and Opioid RMG Together The standardized curves are based on a linear and quadratic term of cumulative receipt of weekly opioid RMG (≥1 day). The survival curves were standardized for baseline covariates and weighted for time-varying confounders. Plus signs indicate censored data.

The association was positive among individuals receiving methadone (ARR RMG ≥4 days, 1.40; 95% CI, 1.37-1.43), buprenorphine-naloxone clients (ARR RMG ≥4 days, 1.56; 95% CI, 1.40-1.73), as well as those receiving other forms of OAT (95% on slow-release oral morphine; ARR for RMG ≥4 days, 1.49; 95% CI, 1.44-1.54). The association of opioid RMG was strong in the weeks during OAT induction (Table 2). Continuous opioid RMG receipt for 6 months reduced the risk of OAT discontinuation by 15% compared with nonrecipients (ARR, 0.85; 95% CI, 0.82-0.88) (Figure 3) among individuals initiating OAT and opioid RMG concurrently. Finally, we found consistent results for alternate definitions of opioid RMG per week. Opioid RMG dispensation up to 2000 MME in a given week increased the probability of OAT receipt in the subsequent week by 31% (ARR, 1.31; 95% CI, 1.29-1.34), whereas receipt of RMG of 2000 MME or more increased the probability of receipt in the subsequent week by 40% (ARR, 1.40; 95% CI, 1.37-1.43) compared with those not receiving opioid RMG. We found consistent results with adjustment for cumulative RMG in the past 4 weeks (see eTable 11 in Supplement 1 for sensitivity analyses). Results of hdPS models were reported throughout, although these findings were highly concordant with those generated through investigator-selected PS adjustment.

## Discussion

In a population-based cohort of people receiving OAT, we found that individuals who were coprescribed opioid RMG had a higher adjusted probability of continued OAT receipt or reengagement compared with those not receiving opioid RMG. These analyses, which adjusted for time-varying confounding and acknowledged the intermittent use of both medications, demonstrated a strong biological gradient and robustness across different population subgroups, with the association especially pronounced when opioids prescribed under the RMG and OAT were initiated concurrently. Although coinitiation was not a requirement of RMG, this finding demonstrates how the guidance was implemented and the associated outcomes.

Our findings should be interpreted with caution. Importantly, decision-making within the physician-client dyad was not observed; therefore, the intended use of RMG prescriptions could only be inferred indirectly. The RMG prescriptions may have been used as temporary patient-led withdrawal management during isolation or as a means of engaging people receiving OAT despite clients’ preferences not to engage in treatment. Nevertheless, we observed a biological gradient in both days prescribed and total weekly doses dispensed, indicative of a therapeutic effect. Ongoing illicit opioid and nonopioid substance use is otherwise commonly reported among OAT clients, with inadequate OAT dosing in the context of a fentanyl-dominated illicit drug supply often cited as a primary determinant of ongoing use.^27,28,29^ In addition to OAT, hydromorphone can diminish withdrawal symptoms for those with high opioid tolerance in the process of titrating OAT to an optimal dose^30^ while reaching a population who have not benefited from OAT alone.^31^

We note that RMG prescribing was implemented on an emergency basis, without new scientific evidence to counter a history of concerns about opioid overprescribing. These factors limited adoption among prescribers and prompted deviations from recommended use. Uptake of RMG was higher among prescribers with larger substance use disorder client loads and peers that previously prescribed RMG, whereas discontinuation was less likely for those whose peers were still prescribing RMG.^32^ Nevertheless, these results suggest one potential use of these RMG opioids as a promising adjunct to OAT. This suggested finding warrants confirmation via a randomized clinical trial, which can optimize hydromorphone dosing and closely monitor medication use, withdrawal symptoms, and other potential adverse events arising from high-dose, combination OAT prescription. On the basis of the Canadian Medical Association’s Code of Ethics,^33^ RMG prescribing cannot justifiably be discontinued, particularly in the midst of a public health emergency.

This study represents a refined approach to evaluating OAT retention as a secondary outcome, as proposed in our protocol.^9^ There were no major deviations from the methods proposed to evaluate this outcome, although given the observed patterns of treatment discontinuation and reengagement, we have focused our efforts on a per protocol approach rather than an intention-to-treat approach. Sensitivity analyses on the induction of OAT as well as medication type and OAT status at RMG initiation were added to further characterize and assess the robustness of the exposure-outcome association. Proposed subgroup analyses on pregnant and parenting people, people with criminal justice system involvement, and people with concurrent mental disorders were underpowered for this evaluation and were thus excluded, as articulated within the protocol.

### Limitations

This study has some limitations. Although we considered both days prescribed and total MME dosing, potential misclassification of exposure was noted above due to lack of uniquely assigned DINs and PINs for RMG prescriptions, and information on withdrawal symptoms and adverse events was absent. In addition, the coding algorithms to identify indicators of alcohol use disorder, tobacco use disorder, and cancer or palliative care using administrative databases were not validated.

We otherwise cannot rule out the potential for unmeasured confounding, although our sensitivity analyses indicated that the magnitudes of estimates observed are unlikely to be reversed by any unmeasured individual or treatment-related factor. Given the high disease burden in people with OUD, the utility of hdPS can meaningfully improve the control of unmeasured confounding on disease severity (eg, skin and soft tissue infection, blood poisoning, and emergency care use) due to additional information being drawn from health administrative data. Finally, this study was executed in a setting with relatively robust access to OAT and other harm reduction interventions where RMG was made available (primarily in BC’s large urban centers; accessibility of OAT and other services is more constrained in rural regions),^34^ and BC has among the highest rates of illicit drug overdose death in North America as a result of the high concentrations of fentanyl and other adulterants in the illicit drug supply.^6,35,36,37^ The extent to which our findings are generalizable to other settings should be considered carefully.

## Conclusions

This population-based cohort study found that coprescription of opioid RMG was associated with substantially higher probability of OAT receipt compared with no opioid RMG receipt. These findings require further investigation to determine safety and isolate the hypothesized therapeutic effects of potential combination formulations of OAT.

## Notes

Supplement 1. **eAppendix 1.** Cohort Definition **eTable 1.** Drug Identification Numbers for Identification of Possible RMG **eTable 2.** Algorithms to Identify Opioid RMG Exposure **eTable 3.** Drug Identification Numbers for Identification of OAT **eTable 4.** Diagnostic Codes Used to Identify Demographic and Medication Use Measures **eTable 5.** Diagnostic Codes Used to Identify Concurrent Chronic Conditions **eAppendix 2.** Selection of Empirical Covariates to Calculate High Dimensional Propensity Scores **eTable 6.** Empirical Covariates Selected for Estimating Effect of Opioid RMG on OAT Receipt in the Month of August 2021 **eAppendix 3.** Monthly Matching Illustration **eFigure 1.** Illustration of Constructing Potential Unexposed Groups for Monthly Matching **eFigure 2.** Distribution of Propensity Scores by Opioid RMG Receipt at Baseline **eTable 7.** Number of Individuals Before and After Matching per Month From Mar/Apr 2020 to Aug 2021 **eTable 8.** Odds Ratios and 95% Confidence Intervals From the Propensity Score Model on the RMG Initiation for January 2021 **eFigure 3.** Distribution of the Inverse Probability Weights for the Marginal Structural Model Among the hdPS-Matched Cohort **eTable 9.** Risk Ratios and 95% Confidence Intervals From the Marginal Structural Outcome Models on OAT Receipt Among People on OAT at Time 0 **eTable 10.** The Effect of Opioid RMG Receipt on OAT Receipt Using hdPS-Matched Cohorts Without Weight Truncation **eFigure 4.** E-Values for the Effect of Opioid RMG on OAT Receipt Using hdPS-Matched Control Among People on OAT at Time 0 **eTable 11.** Sensitivity Analysis on the Alternate Definition of Opioid RMG Receipt for Effect of OAT on Opioid RMG Receipt
eReferences

Supplement 2.
Data Sharing Statement
