# Supplementary material for: Opioid Coprescription Through Risk Mitigation Guidance and Opioid Agonist Treatment Receipt
Source: JAMA Netw Open. 2024 May 15;7(5):e2411389. doi: 10.1001/jamanetworkopen.2024.11389 (PMC11096992; doi:10.1001/jamanetworkopen.2024.11389)
Supplement: Supplement 1. — eAppendix 1. Cohort Definition eTable 1. Drug Identification Numbers for Identification of Possible RMG eTable 2. Algorithms to Identify Opioid RMG Exposure eTable 3. Drug Identification Numbers for Identification of OAT eTable 4. Diagnostic Codes Used to Identify Demographic and Medication Use Measures eTable 5. Diagnostic Codes Used to Identify Concurrent Chronic Conditions eAppendix 2. Selection of Empirical Covariates to Calculate High Dimensional Propensity Scores eTable 6. Empirical Covariates Selected for Estimating Effect of Opioid RMG on OAT Receipt in the Month of August 2021 eAppendix 3. Monthly Matching Illustration eFigure 1. Illustration of Constructing Potential Unexposed Groups for Monthly Matching eFigure 2. Distribution of Propensity Scores by Opioid RMG Receipt at Baseline eTable 7. Number of Individuals Before and After Matching per Month From Mar/Apr 2020 to Aug 2021 eTable 8. Odds Ratios and 95% Confidence Intervals From the Propensity Score Model on the RMG Initiation for January 2021 eFigure 3. Distribution of the Inverse Probability Weights for the Marginal Structural Model Among the hdPS-Matched Cohort eTable 9. Risk Ratios and 95% Confidence Intervals From the Marginal Structural Outcome Models on OAT Receipt Among People on OAT at Time 0 eTable 10. The Effect of Opioid RMG Receipt on OAT Receipt Using hdPS-Matched Cohorts Without Weight Truncation eFigure 4. E-Values for the Effect of Opioid RMG on OAT Receipt Using hdPS-Matched Control Among People on OAT at Time 0 eTable 11. Sensitivity Analysis on the Alternate Definition of Opioid RMG Receipt for Effect of OAT on Opioid RMG Receipt eReferences [file jamanetwopen-e2411389-s001.pdf]

Supplementary Online Content

Min JE, Guerra-Alejos BC, Yan R, et al. Opioid coprescription through risk mitigation guidance and opioid agonist treatment receipt. *JAMA Netw Open*. 2024;7(5):e2411389. doi:10.1001/jamanetworkopen.2024.11389

eAppendix 1. Cohort Definition
eTable 1. Drug Identification Numbers for Identification of Possible RMG
eTable 2. Algorithms to Identify Opioid RMG Exposure
eTable 3. Drug Identification Numbers for Identification of OAT
eTable 4. Diagnostic Codes Used to Identify Demographic and Medication Use Measures
eTable 5. Diagnostic Codes Used to Identify Concurrent Chronic Conditions
eAppendix 2. Selection of Empirical Covariates to Calculate High Dimensional Propensity Scores
eTable 6. Empirical Covariates Selected for Estimating Effect of Opioid RMG on OAT Receipt in the Month of August 2021
eAppendix 3. Monthly Matching Illustration
eFigure 1. Illustration of Constructing Potential Unexposed Groups for Monthly Matching
eFigure 2. Distribution of Propensity Scores by Opioid RMG Receipt at Baseline
eTable 7. Number of Individuals Before and After Matching per Month From Mar/Apr 2020 to Aug 2021
eTable 8. Odds Ratios and 95% Confidence Intervals From the Propensity Score Model on the RMG Initiation for January 2021
eFigure 3. Distribution of the Inverse Probability Weights for the Marginal Structural Model Among the hdPS-Matched Cohort
eTable 9. Risk Ratios and 95% Confidence Intervals From the Marginal Structural Outcome Models on OAT Receipt Among People on OAT at Time 0
eTable 10. The Effect of Opioid RMG Receipt on OAT Receipt Using hdPS-Matched Cohorts Without Weight Truncation
eFigure 4. E-Values for the Effect of Opioid RMG on OAT Receipt Using hdPS-Matched Control Among People on OAT at Time 0
eTable 11. Sensitivity Analysis on the Alternate Definition of Opioid RMG Receipt for Effect of OAT on Opioid RMG Receipt
eReferences

This supplementary material has been provided by the authors to give readers additional information about their work.

eAppendix 1. Cohort Definition

Dispensations of opioid RMG were identified from PharmaNet database. As new DINs were not assigned for RMG, we developed algorithms to identify opioid RMG recipients by applying restrictions to our case searches using prescription data including: drug type prescription history, timing, and a list of keywords from the free-form codes up to 80 characters written in the 'directions for use' variable. From possible opioid RMG dispensation records based on drug type (**Table A1**), we constructed RMG episodes using the 'service date' and 'days supplied' fields in the PharmaNet database. Continuous opioid RMG episodes had no interruptions in prescribed doses lasting ≥ 7 days(1) . An episode with at least one indication of RMG keywords (**Table A2**) was considered as RMG. We used the definition 2 (higher sensitivity, lower specificity) for the exposure variable.

We made some modifications to the protocol (2) in the keyword searching: (i) keywords were searched for any occurrence in the 'directions for use' field, instead of exact words; (ii) more keywords were added (i.e. *safe drug su, safe consum, clean drug supply, fentan* in Definition 1 and *opioid c* (for *opioid craving*), *opioid replacement*, *OAT/s, OAT sa, relieve wi, needed for w, pwd, opioid w, opiate w, first dose w, first dose to be wit, one dose w, 1st w, harm* (excluding *pharm*) in Definition 2) to identify more RMG dispensations; (iii) some keywords were dropped (i.e. *emergency*, *suply, supply, replacement*) because 'emergency supply" (for emergency prescription refills due to expired prescription or out of medication) could be provided not only for RMG dispensation but also for other medications.

We also used PharmaNet to identify individuals who had received opioid agonist treatment (OAT) (**Table A3**).

eTable 1. Drug Identification Numbers for Identification of Possible RMG

**Opioid**	**Drug Identification Numbers DIN/PINs**
Hydromorphone	2364158, 786543, 2225255, 2192144, 2245705, 2337274, 885428, 2319446
Sustained-release oral morphine	2019930, 2019949, 2019957, 2019965, 2177749, 2177757

© 2024 Min JE et al. *JAMA Network Open.*

eTable 2. Algorithms to Identify Opioid RMG Exposure

**Case Definition 1: Higher specificity lower sensitivity**<br>For each chemical type, person NOT on RMG medication in the 2 months prior to March 27, 2020, but prescribed medication from March 27, 2020 with one of the following keywords (i.e. substring) occurred in the directions for use variable: *corona, cov* (excluding *cover*)*, crisis, mitigat, pand* (for *pandemic*)*, pprm, pwm, risk m, riskmitigation, safe s* (for *safe suppl*)*, safer s, safe drug su, safe+supply, safe consum, clean drug supply, fentan.*<br>*Exclusion: "pain" in directions for use variable<br><br>**Case Definition 2: Higher sensitivity, lower specificity**<br>For each chemical type, person NOT on RMG medication in the 2 months prior to March 27, 2020, but prescribed medication from March 27, 2020 with one of the keywords (i.e. substring) occurred in the directions for use variable as listed in definition 1 or any of the following substring in the directions for use variable: *carr, crav, opioid c* (for *opioid craving*)*, del* (for *delivery*)*, distancing, guidan, guideline, illici, interim, isolatio, management, outbreak, overdo, opioid replacement, risk, saf, OAT/s, OAT sa, unwit, no w* (for *no witness*)*, not wi, withd, withrawal, relieve wi, needed for w, pwd, opioid w, opiate w, with wi, witness, wittness, dw, first dose w, first dose to be wit, one dose w, 1st w, harm* (excluding *pharm*).<br>*Exclusion: "pain" in directions for use variable

eTable 3. Drug Identification Numbers for Identification of OAT

DIN/PID* in PharmaNet	Description
999792, 999793, 66999990, 66999991, 66999992, 66999993, 66999997, 66999998, 66999999, 67000000, 67000001, 67000002, 67000003, 67000004, 67000005, 67000006, 67000007, 67000008, 67000009,67000010, 67000011, 67000012, 67000013, 67000014, 67000015, 67000016, 67000017, 67000018, 67000019, 67000020	DIN/PIN for methadone as OAT
2295695, 2295709, 2408090, 2408104, 2424851, 2424878, 2453908, 2453916, 2468085, 2468093, 2502313, 2502321, 2502348, 2502356, 2517175, 2517183	DIN/PIN for buprenorphine/naloxone as OAT
22123346, 22123347, 22123348, 22123349	DIN/PIN for slow-release morphine (Kadian)
2483084, 2483092, 2474921, 9858127, 9858128	DIN/PIN for buprenorphine (Sublocade, Probuphine)
2146126, 22123340, 2469413, 22123357, 66123367	DIN/PIN for Injectable OAT†
786543, 885428	DIN/PIN for t-IOAT (Hydromorphone)

*Drug Identification Numbers (DIN)/Product Identification Numbers(PIN) †Diacetylmorphine or hydromorphone with some restrictions based on prescriber, dispensing pharmacy and/or date.

© 2024 Min JE et al. *JAMA Network Open.*

eTable 4. Diagnostic Codes Used to Identify Demographic and Medication Use Measures

Diseases	Definition and diagnostic code			Reference
Rural region	Rural or remote; categorized by local health authority (LHA) based on population (≤40,000) and geographical consideration (proximity to a larger population centres and health services)			(3)
Income assistance	PharmaCare specialty plan from PharmNet: C (Income Assistance) or receipt of income assistance payment from SDPR			
Unstable housing	ICD-9 from DAD, MSP: V60.0, V60.1, ICD-10 from DAD: Z59.0, Z59.1 or 3 consecutive months of no fixed address from SDPR			(4)
Physician attachment	Low GP use: visited any GP (or nurse practitioner) less than three times in the past year; single GP >50%: had three or more GP visits and more than 50% of the visits were to a single GP when receiving core primary care services (MSP).			(5)
Overdose acute care visits	ICD-10 from DAD or NACRS: T40.0, T40.1, T40.2, T40.3, T40.4, T40.5, T40.6, T43.6			
Opioid dispensation for pain	Dispensation of codeine, fentanyl, hydromorphone, morphine, oxycodone, sufentanil from PharmaNet			
Benzodiazepine dispensation	Dispensation of alprazolam, bromazepam, chlordiazepoxide, clobazam, clonazepam, clorazepate, diazepam, flurazepam, ketazolam, lorazepam, midazolam, nitrazepam, oxazepam, temazepam, triazolam from PharmaNet			
OAT guideline compliance (maximum daily dose per week)		Week <4	≥Week 4	(6-9)
	Methadone	Any	≥80mg	
	Buprenorphine/naloxone	Any	≥16mg/4mg	
	Slow-release oral morphine	Any	≥600mg	
	Injectable hydromorphone	Any	≥200mg	
	Injectable diacetylmorphine	Any	≥400mg	
	Extended-release buprenorphine	Any	100mg or 300mg Sublocade per month	
	Tablet injectable OAT (hydromorphone)*	Any	≥80mg	

ICD: International Classification of Diseases; DAD: Discharge Abstract Database; MSP: Medical Service Plan; SDPR: Social Development and Poverty Reduction; GP: general practitioner. * based on median value

© 2024 Min JE et al. *JAMA Network Open.*

eTable 5. Diagnostic Codes Used to Identify Concurrent Chronic Conditions

Diseases	Diagnostic code	References	Validation
Charlson comorbidity index	Based on 17 CCI groups using enhanced ICD-9-CM and ICD-10 codes from hospitalization records in DAD in the past year	(10), (11)	Yes, predictive validity on in-hospital mortality(10) and validity of the coding algorithms(12) among in-patients aged 18 or older in Calgary Health Region
Chronic Disease score	Based on 29 categories using AHFS from medication dispensation records in PharmaNet in the past year	(13)	Yes, predictive validity on hospitalization and death among people aged 18 or older in Western Washington State(13)
Severe mental health disorder	Includes major depressive disorder, bipolar disorder, and schizophrenia		
Major depressive disorder¶	ICD-9 from DAD, MSP: 296.2, 296.3<br>ICD-10 from DAD, NACRS: F32.1, F32.2, F32.9, F33.1, F33.2, F33.9	(14), (15)	Yes, using ICD-9 (296.2, 296.3, 300.4, 311)(15-17)
Bipolar disorder¶	ICD-9 from DAD, MSP: 296.4, 296.5, 296.6, 296.7, 296.8<br>ICD-10 from DAD, NACRS: F31	(18)	Yes, using the ICD-9 (296.x (0,1,2,3,4,8,9)) and ICD-10 (F30-31) from hospital discharge records in Scandinavia (19, 20)
Schizophrenia±	ICD-9 from DAD, MSP: 295<br>ICD-10 from DAD, NACRS: F20.0, F20.1, F20.2, F20.5, F20.8, F20.9	(14), (15), (21)	Yes, using ICD-10 (F20, F25) and ICD-9 (295) in Canada (15, 21, 22)
HIV*	ICD-9 from DAD and MSP: 042-044, 079.53, 795.8, V08; ICD-10 from DAD and NACRS: B20-B24, B97.35, F02.4, O98.7, Z21; MSP fee item: 13015, 13105, 33645, 36370	(23)	Yes, using ICD-9 (042-044, V08) and ICD-10 (B20-B24, R75, Z21) in British Columbia, Canada(23)
Hepatitis C virus*	ICD-9 from DAD and MSP: 070.4, 070.5, 070.7; ICD-10 from DAD and NACRS: B17.1, B18.2, B19.2; AHFS category: 8:18.40	(24), (25), (26)	Yes, using the ICD-10-CM in a health care system in Taiwan(27)
Alcohol use disorder*	ICD-9 from DAD and MSP: 291, 303, 305.0, 357.5, 425.5, 535.3, 571.0-571.3, 655.4, V65.42; ICD-10 from DAD and NACRS: F10, Z50.2, Z71.4, Z72.1, G31.2, G62.1, G72.1, I42.6, K29.2, K70, K86.0, O35.4; DIN: 2293269, 2158655, 2213826, 2444275, 2451883, 2534, 2542, 2041375, 2041391, 66124089, 66124085, 66124087; indication of alcohol use disorder during the pregnancy from BCPDR	(28), (29), (30)	No
Substance use disorder*‡	ICD-9 from DAD and MSP: 292, 304.x (1-6,8,9), 305.x (2-4,6-9), 648.3, 655.5, 967, 969.x (4,6,7), 970, E851, E852, E853.2, E854.x (1,2,3); ICD-10 from DAD and NACRS: F12-F16, F19, X42, X62, Y12, T40.5, T40.7-T40.9, T42.4, T43.6, Z50.3, Z71.5, Z72.2; indication of substance use during the pregnancy from BCPDR	(10, 28)	Yes, using ICD-10 (F11-F16, F18, F19, Z71.5, Z72.2) and ICD-9-CM (292, 304, 305.2-305.9, V65.42) in-patients aged 18 or older in Calgary Health Region(12)

© 2024 Min JE et al. *JAMA Network Open.*

Chronic pain*§	ICD-9 from DAD and MSP: 338.2, 338.4, 307.80, 307.89, 338.0, 719.41, 719.45-719.47, 719.49, 720.0, 720.2, 720.9, 721.0-721.4, 721.6, 721.8, 721.9, 722, 723.0, 723.1, 723.3-723.9, 724.0-724.6, 724.70, 724.79, 724.8, 724.9, 729.0-729.2, 729.4, 729.5, 350, 352-357, 344.0, 344.1, 997.0, 733.0, 733.7, 733.9, 781; ICD-10 from DAD and NACRS: F45.4, G89.0, G89.2, G89.4, M08.1, M25.50, M25.51, M25.55-M25.57, M43.2-M43.6, M45, M46.1, M46.3, M46.4, M46.9, M47, M48.0, M48.1, M48.8, M48.9, M50.8, M50.9, M51, M53.1-M53.3, M53.8, M53.9, M54, M60.8, M60.9, M63.3, M79.0-M79.2, M79.6, M79.7, M96.1, G50, G52-G64, G82, G97, M89, R29	, (31), (32, 33)	Yes, using the ICD-9 codes at community health center in Connecticut, USA(31)
Tobacco use disorder*	ICD-9 from DAD and MSP: 305.1, V15.82. ICD-10 from DAD: F17, O99.33, T65.2, Z71.6, Z72.0, Z87.8. PharmaNet: indication of nicotine replacement therapy	(34)	No
Cancer*	ICD-9 from DAD and MSP: 140-208. ICD-10 from DAD and NACRS: C00-C97.	(35, 36)	No
Palliative care*	MSP fee-item: 127, 14063, 36640, 38006, 96163, 96169, 96963, 96969, 114, 115, 9910, 9911, 9912, 13114, 13334, 36355, 36356. ICD-9 from DAD and MSP: V66.7; ICD-10 from DAD: Z51.5. Any PharmaNet records under the PharmaCare plan for palliative care (Plan P).	(37)	No

ICD: International Classification of Diseases; DAD: Discharge Abstract Database; NACRS: National Ambulatory Care Reporting System; MSP: Medical Service Plan; AHFS: American Hospital Formulary Service from PharmaNet; DIN: drug identification number; BCPDR: BC Perinatal data registry. *To minimize misclassification due to errors in the coding of physician billing records, we applied a case-finding algorithm based on the presence of at least 1 hospitalization, ED visit, BCPDR record, more than 3 physician billing records, or medication receipt for alcohol use disorder; ¶ case-finding algorithm: ≥1 hospitalization, ≥1 ED visits, or ≥2 physician visits in one year; ± case-finding algorithm: ≥1 hospitalization, ≥1 ED visits, or ≥2 physician visits at least 30 days apart in two years; ‡Any indication of non-opioid drug use, poisoning (accidental or intentional), or substance use counselling or rehab, excluding alcohol use disorder; §non-cancer

© 2024 Min JE et al. *JAMA Network Open.*

eAppendix 2. Selection of Empirical Covariates to Calculate High Dimensional Propensity Scores

We applied the high-dimensional propensity score (hdPS) algorithm by month to generate 50 empirically-derived covariates specific to each outcome from administrative databases(38). We used 5 data dimensions: 4-digit ICD-10 codes and 5-digit inpatient procedure codes from DAD (hospitalizations), 3-digit ICD-9 codes and 5-digit fee item from MSP (physician visits), and drug dispensed (8-digit TC5; excluding opioid RMG) from Pharmanet databases during a 6-mongth prior to baseline. In order to identify candidate empirical covariates, we divided each code into three binary variables based on frequency: code occurred ≥ 1 times (“once”), ≥ median number of times (“sporadic”), and ≥ 75th percentile number of times (“frequent”). Then, we chose top 100 most prevalent code in each data dimensions based on frequency of code occurrence, resulting 5 dimensions * 100 codes * 3 binary variables=1,500 covariates. Among those covariates, we included the top 50 covariates based on apparent relative risk to combine information from all 5 data dimensions(38). **eTable 6** lists the 50 selected covariates for analysis of effect of opioid RMG on OAT measured on month of August 2021.

© 2024 Min JE et al. *JAMA Network Open.*

eTable 6. Empirical Covariates Selected for Estimating Effect of Opioid RMG on OAT Receipt in the Month of August 2021

Dimension	Diagnosis code	Frequency	Code Description
ICD-10 from DAD	B95.6	Once	Staphylococcus aureus as the cause of diseases classified to other chapters
ICD-10 from DAD	D50.9	Once	Iron deficiency anaemia, unspecified
ICD-10 from DAD	F10.2	Once	Mental and behavioural disorders due to use of alcohol: dependence syndrome
ICD-10 from DAD	F10.3	Once	Mental and behavioural disorders due to use of alcohol withdrawal state
ICD-10 from DAD	F11.2	Once	Mental and behavioural disorders due to use of opioids: dependence syndrome
ICD-10 from DAD	L03.1	Once	Cellulitis of other parts of limb
ICD-10 from DAD	U07.5	Frequent	Personal history of COVID-19
ICD-10 from DAD	U07.5	Once	Personal history of COVID-19
ICD-10 from DAD	U82.1	Once	Resistance to methicillin
ICD-10 from DAD	U98.9	Once	Unspecified place of occurrence
ICD-10 from DAD	Z22.3	Once	Carrier of other specified bacterial diseases
ICD-10 from DAD	Z59.0	Once	Homelessness
ICD-9 from MSP	038	Once	septicaemia
ICD-9 from MSP	038	Sporadic	septicaemia
ICD-9 from MSP	038	Frequent	septicaemia
ICD-9 from MSP	285	Frequent	other and unspecified anaemias
ICD-9 from MSP	293	Frequent	transient organic psychotic conditions
ICD-9 from MSP	682	Once	other cellulitis and abscess
ICD-9 from MSP	780	Frequent	general symptoms
ICD-9 from MSP	780	Once	general symptoms
ICD-9 from MSP	965	Once	poisoning by analgesics, antipyretics and antirheumatics
Fee item from MSP	100	Once	visit in office (age 2 - 49)
Fee item from MSP	108	Sporadic	hospital visit
Fee item from MSP	108	Frequent	hospital visit
Fee item from MSP	108	Once	hospital visit
Fee item from MSP	109	Once	acute care hospital admission visit
Fee item from MSP	116	Once	consultation, special in-hospital
Fee item from MSP	1202	Once	call-out charge/saturday, sunday, or stat holiday
Fee item from MSP	1205	Once	surcharge - nonoperative - evening
Fee item from MSP	1207	Frequent	surcharge - nonoperative/weekend and stat/holiday
Fee item from MSP	1207	Once	surcharge - nonoperative/weekend and stat/holiday
Fee item from MSP	13008	Frequent	community based gp: hospital visit
Fee item from MSP	13008	Sporadic	community based gp: hospital visit
Fee item from MSP	13008	Once	community based gp: hospital visit

© 2024 Min JE et al. *JAMA Network Open.*

Fee item from MSP	15039	Once	gp poc testing for opioid agonist treatment
Fee item from MSP	1813	Once	level iii emergency care - day
Fee item from MSP	1832	Frequent	level ii emergency care - night
Fee item from MSP	1832	Once	level ii emergency care - night
Fee item from MSP	1833	Once	level iii emergency care - night
Fee item from MSP	1843	Once	level iii emergency care - sat, sun or stat hol
Fee item from MSP	615	Once	hospital/institution inpatient or home visit
Fee item from MSP	650	Once	psychotherapy indiv.(hosp or institut) per 1/2 hr
Fee item from MSP	8692	Once	tomography-head scan double scan or 2 planes
Fee item from MSP	90465	Once	blood film review
Drug from PNet	08120606	Once	cephalexin
Drug from PNet	28080805	Sporadic	hydromorphone
Drug from PNet	28080805	Frequent	hydromorphone
Drug from PNet	28080805	Once	hydromorphone
Drug from PNet	28080809	Once	morphine
Drug from PNet	28080809	Sporadic	morphine

ICD: International Classification of Diseases (9 or 10th Revision); DAD: Discharge Abstract Database for hospitalisations; MSP: Medical Services Plan for physician billing records; PNet: PharmaNet for community pharmacy dispensations. * frequency: ≥ 1 times (“once”), ≥ median number of times (“sporadic”), and ≥ 75th percentile number of times (“frequent”).

© 2024 Min JE et al. *JAMA Network Open.*

eAppendix 3. Monthly Matching Illustration

Weekly observations were constructed from March 27, 2020 or the first week of OAT receipt, whichever occurred later. Monthly matching was done based on the propensity scores estimated at the first week of opioid RMG receipt among ever RMG recipients and the first week on OAT of each month prior to the month of RMG initiation. We executed matching without replacement using the nearest neighbor matching using a SAS macro version 2 available at: www.drugepi.org.(39) The pairwise nearest neighbour matching computed the best matches based on the distance between each pair of individuals, which would give consistent results independent of any ordering of individuals.(40) We used a caliper of width equal to 0.2 of the standard deviation of the logit of the propensity score(41)

Once selected as an unexposed group in an early month, the person cannot be selected again in the unexposed group in the later months. However, exposed individuals can be selected as an unexposed group prior to their first RMG receipts, and the observations were censored at the RMG receipt.

eFigure 1. Illustration of Constructing Potential Unexposed Groups for Monthly Matching

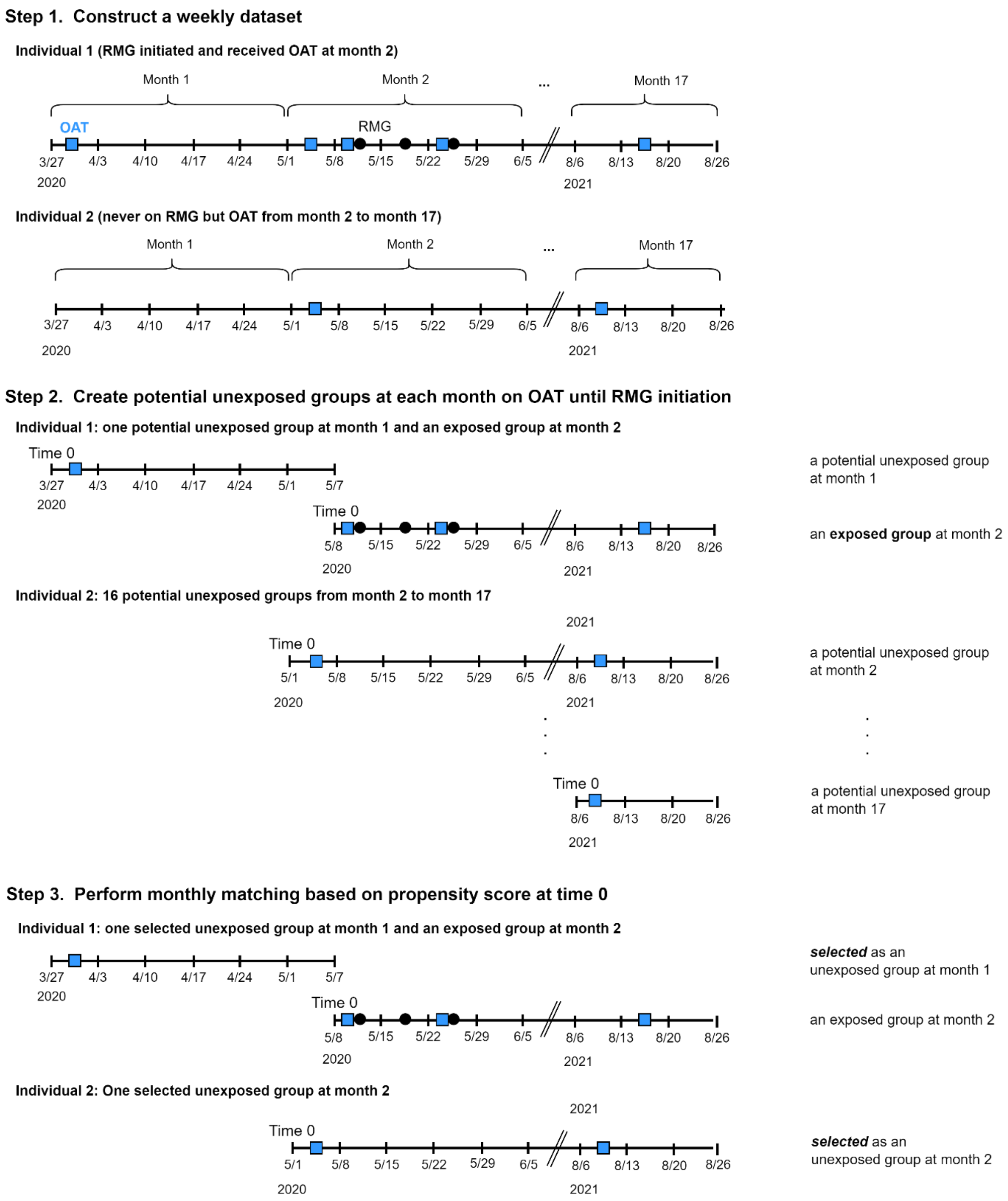

A black circle indicates opioid RMG receipt per week, and a blue square indicates OAT receipt per week.

© 2024 Min JE et al. *JAMA Network Open.*

eFigure 2. Distribution of Propensity Scores by Opioid RMG Receipt at Baseline

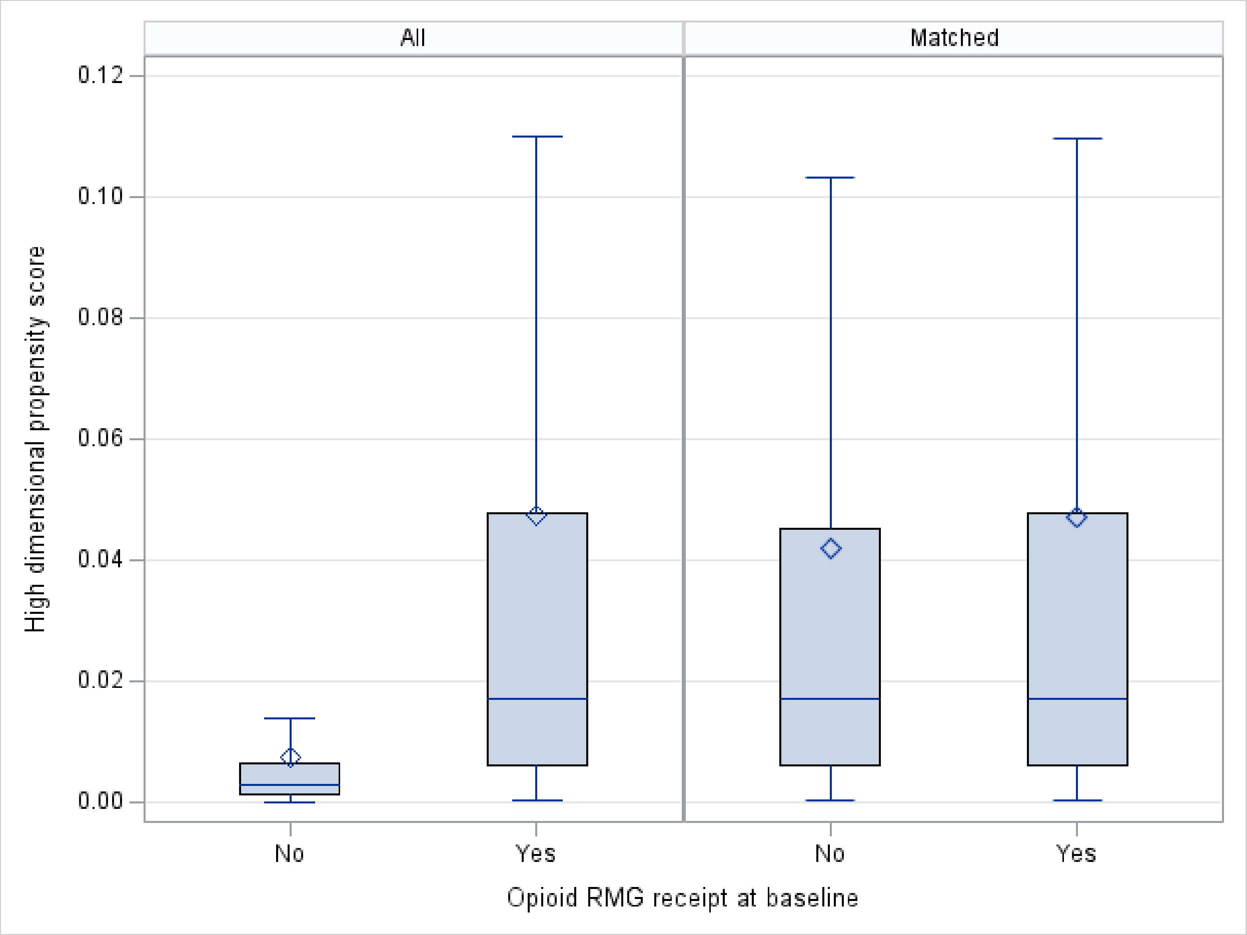

Each box plot displays the mean (as a diamond), median (middle horizontal bar) and the first and third quartiles (border horizontal bars). Vertical lines were drawn from the box to the most extreme point that was less than or equal to 1.5 times the intra-quartile range. The two distributions were well-balanced for the matched cohort at baseline.

© 2024 Min JE et al. *JAMA Network Open.*

eTable 7. Number of Individuals Before and After Matching per Month From Mar/Apr 2020 to Aug 2021

Month Number	Month	Before matching		After matching	
		Opioid RMG receipt		Opioid RMG receipt	
		Yes	No	Yes	No
1	Mar/Apr 2020	271	21,359	271	271
2	May 2020	309	20,747	308	308
3	June 2020	245	20,326	245	245
4	July 2020	278	20,121	278	278
5	Aug. 2020	257	19,809	257	257
6	Sep. 2020	315	19,531	315	315
7	Oct. 2020	394	19,608	394	394
8	Nov. 2020	280	19,540	280	280
9	Dec. 2020	249	19,456	249	249
10	Jan. 2021	423	19,157	421	421
11	Feb. 2021	296	19,410	296	296
12	Mar. 2021	220	19,274	220	220
13	April 2021	277	19,138	277	277
14	May 2021	191	19,195	191	191
15	June 2021	202	19,078	202	202
16	July 2021	297	18,674	297	297
17	Aug. 2021	132	18,558	132	132
Overall		4,636	31,711	4,633	4,633

© 2024 Min JE et al. *JAMA Network Open.*

eTable 8. Odds Ratios and 95% Confidence Intervals From the Propensity Score Model on the RMG Initiation for January 2021

Characteristics	Odds ratio (95% CI)	P-value
Sex: female vs. male	1.0 (0.8-1.2)	0.680
Age: 18-29 vs. 50+	1.6 (1.1-2.3)	0.021
30-39	1.8 (1.3-2.4)	0.001
40-49	1.4 (1.0-2.0)	0.033
Region: rural vs. urban	1.0 (0.7-1.4)	0.827
Vancouver or South/Central Vancouver Island	1.9 (1.5-2.3)	<.0001
Receipt of income assistance in the past year^	2.6 (1.9-3.5)	<.0001
Unstable housing in the past year†	1.6 (1.3-2.1)	0.000
OAT in the last week: none vs. OAT guideline noncompliant	23.3 (16.2-33.4)	<.0001
OAT guideline compliant ±	1.9 (1.3-2.6)	<.0001
Years since OUD diagnosis: <5 vs. ≥10	1.2 (0.9-1.6)	0.102
5-9	1.0 (0.7-1.3)	0.307
Charlson Comorbidity Index (CCI) > 0	0.9 (0.6-1.5)	0.704
Chronic Disease Score (CDS)	1.1 (0.9-1.2)	0.449
Overdose-related acute care visits in the past month	2.1 (1.3-3.4)	0.004
Other substance use disorder (ever)*§	1.5 (1.0-2.2)	0.028
Alcohol use disorder (ever)*	1.2 (0.9-1.5)	0.194
Serious mental disorder(ever) * ¶	0.9 (0.7-1.2)	0.642
HIV (ever) *	0.7 (0.4-1.2)	0.215
Hepatitis C (ever)*	1.3 (1.0-1.8)	0.062
Chronic pain (past year)*	0.8 (0.6-1.0)	0.043
Tobacco use disorder (past year)*	0.7 (0.5-0.9)	0.010
Any cancer or palliative care in the past year*	0.9 (0.6-1.3)	0.547
Incarcerated in the past year‡	1.3 (0.8-1.9)	0.266
Physician attachment: low GP use or single GP ≤50% vs. >50%	2.0 (1.6-2.5)	<.0001
Dispensations opioid other than OAT in the past 60 days	3.4 (2.6-4.5)	<.0001
Dispensations of benzo medication in the past 60 days	0.5 (0.3-0.9)	0.023
Dispensations of stimulant RMG	0.9 (0.5-1.7)	0.789

Abbreviations: OAT: opioid agonist treatment; Opioid RMG: opioid risk mitigation guidance prescriptions (hydromorphone tablets or sustained-release oral morphine); PS: propensity score; hdPS: high dimensional propensity score, using also algorithm-selected covariates. ^ Plan C from PharmaNet or income assistance record from Ministry of Social Development and Poverty Reduction (SDPR). † ICD9: V60.0, V60.1; ICD10: Z59.0, Z59.1 from MSP or DAD or 3 consecutive records of no fixed address from SDPR. ± based on the maximum daily dose per week from the 4th week of OAT episode initiation (≥80mg for methadone; ≥16mg/3mg for buprenorphine/naloxone; ≥600mg for slow-release oral morphine; ≥200mg for injectable hydromorphone; ≥400mg for injectable diacetylmorphine; 100mg or 300mg per month for extended-release buprenorphine; ≥80mg for tablet injectable hydromorphone). ‡ Admission to BC correctional facilities. * See Table A5 for ICD-9/ICD-10 codes used to identify concurrent chronic conditions. § Excludes opioid use disorder and alcohol use disorder. ¶ Includes major depressive disorder, bipolar disorder, and schizophrenia.

© 2024 Min JE et al. *JAMA Network Open.*

eFigure 3. Distribution of the Inverse Probability Weights for the Marginal Structural Model Among the hdPS-Matched Cohort

Panel A. Before truncation: mean=1.1; std dev=4.4

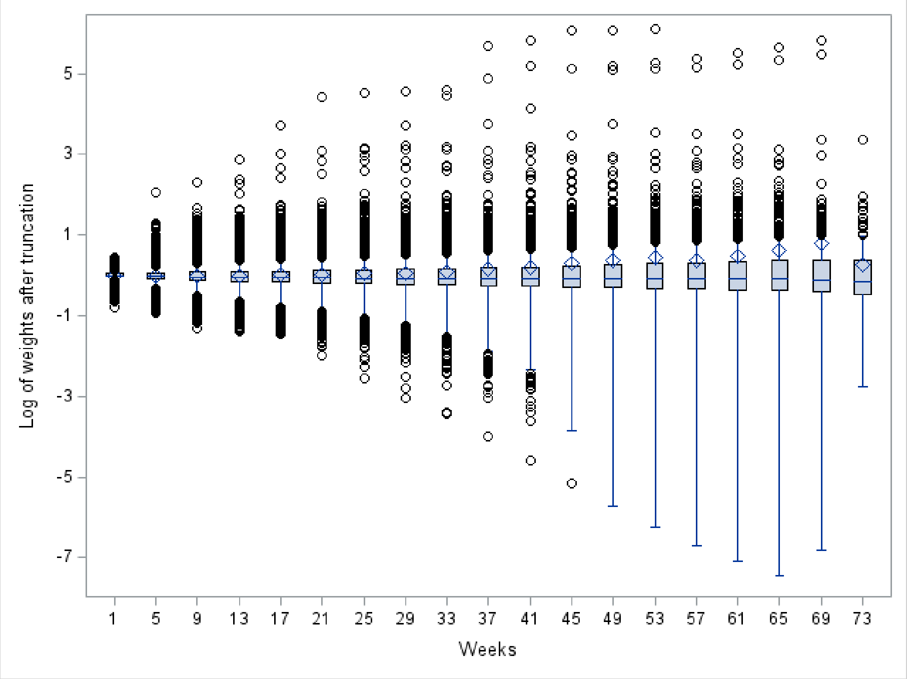

Panel B. After truncation at the first and 99th percentiles: mean=1.0; std dev=0.4

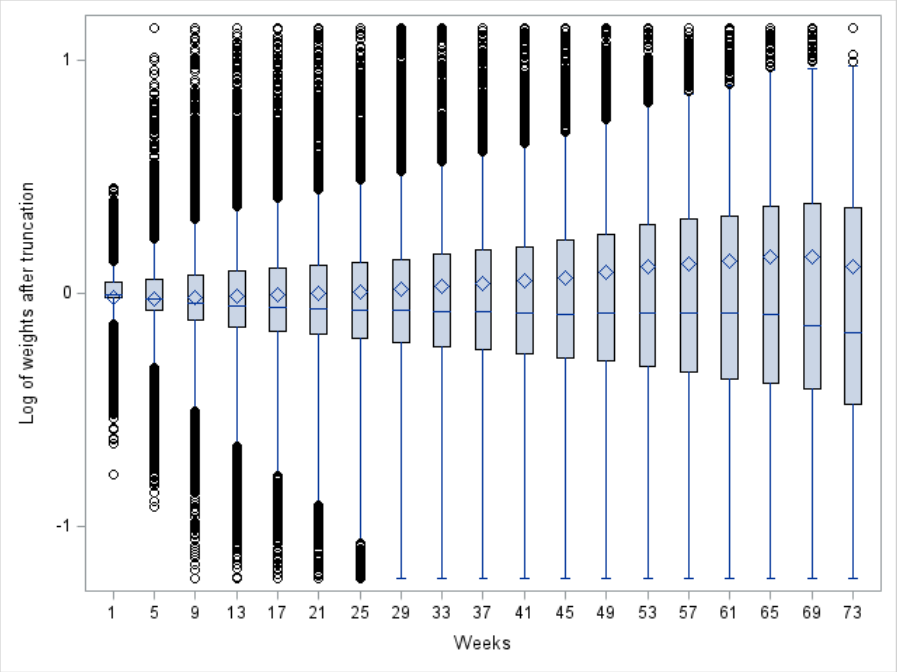

© 2024 Min JE et al. *JAMA Network Open.*

eTable 9. Risk Ratios and 95% Confidence Intervals From the Marginal Structural Outcome Models on OAT Receipt Among People on OAT at Time 0

Characteristics [a]	PS-matched cohort	hdPS-matched cohort
Opioid RMG: 1-3 days vs. none	1.26 (1.24-1.29)	1.27 (1.25-1.30)
≥ 4 days	1.45 (1.42-1.47)	1.46 (1.43-1.49)
Sex: female vs. male	0.92 (0.89-0.94)	0.93 (0.91-0.95)
Age	1.00 (1.00-1.00)	1.00 (1.00-1.00)
Region: rural vs. urban	0.99 (0.95-1.03)	0.97 (0.93-1.01)
Vancouver or South/Central Vancouver Island	0.98 (0.96-1.01)	0.99 (0.97-1.02)
Receipt of income assistance in the past year^ [b]	0.97 (0.94-1.00)	0.96 (0.93-0.99)
Unstable housing in the past year† [b]	0.93 (0.91-0.96)	0.94 (0.91-0.96)
OAT in the last week [b]: none vs. OAT guideline noncompliant	0.73 (0.70-0.75)	0.74 (0.71-0.77)
OAT guideline compliant ±	0.96 (0.94-0.99)	0.96 (0.93-0.99)
Years since OUD diagnosis: <5 vs. ≥10	0.93 (0.90-0.96)	0.94 (0.91-0.97)
5-9	0.96 (0.92-0.99)	0.97 (0.94-1.01)
Charlson Comorbidity Index (CCI) > 0 [b]	0.99 (0.95-1.04)	0.99 (0.94-1.03)
Chronic Disease Score (CDS) [b]	1.03 (1.01-1.05)	1.02 (1.01-1.04)
Overdose-related acute care visits in the past month [b]	1.01 (0.95-1.06)	1.01 (0.96-1.07)
Other substance use disorder (ever)*§ [b]	1.02 (0.98-1.07)	1.02 (0.97-1.07)
Alcohol use disorder (ever)* [b]	1.00 (0.97-1.02)	1.00 (0.97-1.02)
Serious mental disorder(ever) * ¶ [b]	1.04 (1.01-1.07)	1.05 (1.02-1.08)
HIV (ever) * [b]	0.96 (0.91-1.01)	0.95 (0.91-1.00)
Hepatitis C (ever)* [b]	1.04 (1.01-1.07)	1.06 (1.03-1.09)
Chronic pain (past year)* [b]	1.01 (0.99-1.04)	0.99 (0.96-1.02)
Tobacco use disorder (past year)* [b]	1.07 (1.04-1.10)	1.07 (1.04-1.10)
Any cancer or palliative care in the past year* [b]	0.99 (0.95-1.03)	0.99 (0.95-1.03)
Incarcerated in the past year‡ [b]	0.88 (0.84-0.93)	0.88 (0.83-0.93)
Physician attachment: low GP use or single GP ≤50% vs. >50% [b]	0.92 (0.90-0.94)	0.93 (0.91-0.95)
Dispensations opioid other than OAT in the past 60 days [b]	0.97 (0.94-1.00)	0.97 (0.94-1.00)
Dispensations of benzo medication in the past 60 days [b]	1.01 (0.96-1.07)	1.00 (0.95-1.06)

Abbreviations: OAT: opioid agonist treatment; Opioid RMG: opioid risk mitigation guidance prescriptions (hydromorphone tablets or sustained-release oral morphine); PS: propensity score; hdPS: high dimensional propensity score, using also algorithm-selected covariates. ^ Plan C from PharmaNet or income assistance record from Ministry of Social Development and Poverty Reduction (SDPR). † ICD9: V60.0, V60.1; ICD10: Z59.0, Z59.1 from MSP or DAD or 3 consecutive records of no fixed address from SDPR. ± based on the maximum daily dose per week from the 4th week of OAT episode initiation (≥80mg for methadone; ≥16mg/3mg for buprenorphine/naloxone; ≥600mg for slow-release oral morphine; ≥200mg for injectable hydromorphone; ≥400mg for injectable diacetylmorphine; 100mg or 300mg per month for extended-release buprenorphine; ≥80mg for tablet injectable hydromorphone). ‡ Admission to BC correctional facilities. * See Table A5 for ICD-9/ICD-10 codes used to identify concurrent chronic conditions. § Excludes opioid use disorder and alcohol use disorder. ¶ Includes major depressive disorder, bipolar disorder, and schizophrenia. [a] Baseline variable at time0 except for opioid RMG variables. [b] time-dependent variables used to calculate the denominator of weights in the marginal structural model.

© 2024 Min JE et al. *JAMA Network Open.*

eTable 10. The Effect of Opioid RMG Receipt on OAT Receipt Using hdPS-Matched Cohorts Without Weight Truncation

	N [b]	Risk ratio (95% CI) for RMG vs. no RMG	
		RMG 1-3 days	RMG ≥ 4 days
Primary analysis	4,633	1.24 (1.16-1.32)	1.50 (1.45-1.56)
Subgroup analyses [a]:			
People initiating OAT and opioid RMG concurrently	2,352	1.51 (1.41-1.63)	2.02 (1.92-2.13)
During OAT induction (up to 4 weeks)	2,352	1.06 (1.03-1.09)	1.42 (1.39-1.45)
People initiating OAT prior to opioid RMG initiation	2,281	1.07 (1.05-1.09)	1.14 (1.12-1.17)
People initiating/on methadone	2,859	1.39 (1.19-1.63)	1.56 (1.37-1.78)
People initiating/on buprenorphine/naloxone	427	1.38 (1.24-1.54)	1.64 (1.47-1.83)
People initiating/on other forms of OAT	1,348	1.28 (1.22-1.34)	1.63 (1.50-1.76)

OAT: opioid agonist treatment; Opioid RMG: opioid risk mitigation guidance prescriptions (hydromorphone tablets or sustained-release oral morphine); hdPS: high dimensional propensity score matching, using investigated-selected covariates and algorithm-selected covariates. [b] Based on RMG initiation week for the exposure group and the matched unexposed group; [b] Number of individuals on RMG, matched with unexposed group. Marginal structural model with inverse probability of treatment weights. Controlling for: age, sex, rurality of residence, service access (Vancouver or South/Central Vancouver Island vs. others), income assistance receipt - past 12m, prior-week OAT dispensation (none, suboptimal, optimal), unstable hosing – past 2 m, years since the first OUD indication (<5, 5-9, ≥10), overdose-related acute care visits – past 30 days, Charlson Comorbidity index (>0), Chronic Disease Score, any prior indication of disorder for substance use, alcohol use, mental health, HIV, Hepatitis C, chronic pain – past 12m, tobacco use - past 12m, any cancer/palliative care – past 12m, incarcerated – past 12m, physician attachment – past 12m, opioid dispensation other than OAT prescription – past 60 days, benzodiazepine dispensation – past 60 days, week of follow-up (linear & quadratic), and calendar month of time zero from 1 (Mar/April 2020) to 17 (Aug 2021) (linear & quadratic).

© 2024 Min JE et al. *JAMA Network Open.*

eFigure 4. E-Values for the Effect of Opioid RMG on OAT Receipt Using hdPS-Matched Control Among People on OAT at Time 0

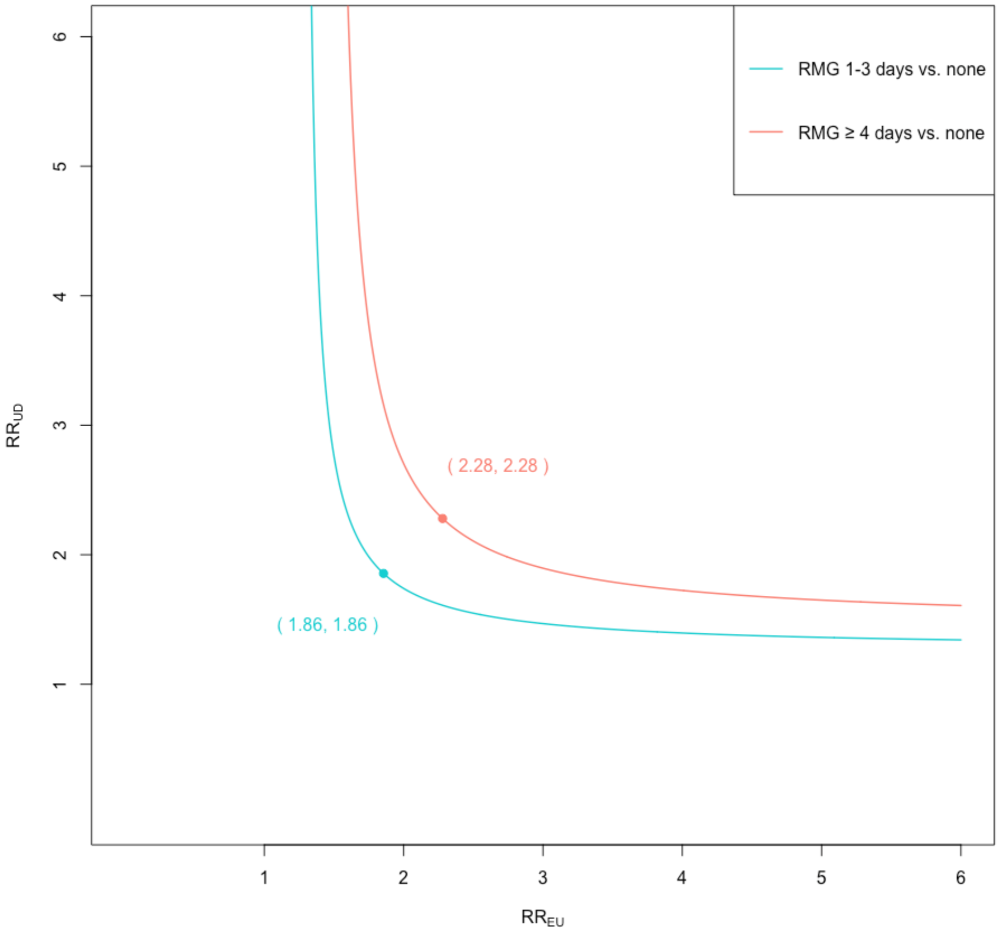

Legend: OAT: opioid agonist treatment; RMG: opioid risk mitigation guidance prescriptions (hydromorphone tablets or sustained-release oral morphine); RR_{UD}: risk ratio relating unmeasured confounders to OAT receipt; RR_{EU}: risk ratio relating to opioid RMG to unmeasured confounders; hdPS: high-dimensional propensity score matching, using algorithm-selected covariates.

© 2024 Min JE et al. *JAMA Network Open.*

eTable 11. Sensitivity Analysis on the Alternate Definition of Opioid RMG Receipt for Effect of OAT on Opioid RMG Receipt

RMG definition	Risk ratio (95% CI) for RMG vs. no RMG	
	Low RMG	High RMG
Primary analysis: low: RMG 1-3; high: ≥ 4 days	1.27 (1.25-1.30)	1.46 (1.43-1.49)
Sensitivity analyses:		
Low: RMG 1-5; high: ≥ 6 days	1.32 (1.29-1.35)	1.47 (1.44-1.50)
Low: RMG <2000 MME, high: ≥ 2000 MME	1.31 (1.29-1.34)	1.40 (1.37-1.43)
Low: current RMG 1-3; high: ≥ 4 days, adjusting for cumulative RMG [a] in past 4 weeks	1.24 (0.22-1.26)	1.39 (1.36-1.41)

Legend: OAT: opioid agonist treatment; RMG: opioid risk mitigation guidance prescriptions (hydromorphone tablets or sustained-release oral morphine); PS: propensity score matching, using investigator-selected covariates; hdPS: high dimensional propensity score matching, using also algorithm-selected covariates; MME: Morphine Equivalent weekly dosage dispensed. Marginal structural model with inverse probability of treatment weights. Controlling for: age, sex, rurality of residence, service access (Vancouver or South/Central Vancouver Island vs. others), income assistance receipt - past 12m, prior-week OAT dispensation (none, suboptimal, optimal), unstable hosing – past 2 m, years since the first OUD indication (<5, 5-9, ≥10), overdose-related acute care visits – past 30 days, Charlson Comorbidity index (>0), Chronic Disease Score, any prior indication of disorder for substance use, alcohol use, mental health, HIV, Hepatitis C, chronic pain – past 12m, tobacco use - past 12m, any cancer/palliative care – past 12m, incarcerated – past 12m, physician attachment – past 12m, opioid dispensation other than OAT prescription – past 60 days, benzodiazepine dispensation – past 60 days, week of follow-up (linear & quadratic), and month of time zero from 1 to 17 (a linear & quadratic); [a] having at least a day of opioid RMG per week (a linear & quadratic terms).

© 2024 Min JE et al. *JAMA Network Open.*

eReferences

1. British Columbia Centre on Substance Use. Risk Mitigation in the Context of Dual Public Health Emergencies: Update to Interim Clinical Guidance. 2022 [Available from: https://www.bccsu.ca/wp-content/uploads/2022/02/Risk-Mitigation-Guidance-Update-February-2022.pdf.
2. Nosyk B, Slaunwhite A, Urbanoski K, Hongdilokkul N, Palis H, Lock K, et al. Evaluation of risk mitigation measures for people with substance use disorders to address the dual public health crises of COVID-19 and overdose in British Columbia: a mixed-method study protocol. BMJ Open. 2021;11(6):e048353.
3. British Columbia Ministry of Health. B.C. Health System Strategy Geographic Service Areas 2016 [Available from: https://www2.gov.bc.ca/assets/gov/health/about-bc-s-health-care-system/health-priorites/geographic-service-areas.docx.
4. Peterson R, Gundlapalli AV, Metraux S, Carter ME, Palmer M, Redd A, et al. Identifying Homelessness among Veterans Using VA Administrative Data: Opportunities to Expand Detection Criteria. PLoS One. 2015;10(7):e0132664.
5. Provincial Health Services Authority. Identifying determinants for hospitalization based on healthcare utilization records. 2008 [Available from: http://www.bccdc.ca/pop-public-health/Documents/Identifying%20determinants%20for%20hospitalization%20based%20on%20healthcare%20utilization%20records%20%282008%29.pdf.
6. British Columbia Centre on Substance Use. Sublocade (Extended-Release Buprenorphine) Information 2022 [Available from: https://www.bccsu.ca/wp-content/uploads/2022/01/Bulletin-Sublocade-Jan2022.pdf.
7. British Columbia Centre on Substance Use. Opioid Use Disorder: Practice Update 2022 [Available from: https://www.bccsu.ca/wp-content/uploads/2022/02/Opioid-Use-Disorder-Practice-Update-February-2022.pdf.
8. British Columbia Centre on Substance Use and B.C. Ministry of Health. A Guideline for the Clinical Management of Opioid Use Disorder. 2017.
9. British Columbia Centre on Substance Use and B.C. Ministry of Health. Guidance for Injectable Opioid Agonist Treatment for Opioid Use Disorder. 2021.
10. Quan H, Sundararajan V, Halfon P, Fong A, Burnand B, Luthi JC, et al. Coding algorithms for defining comorbidities in ICD-9-CM and ICD-10 administrative data. Med Care. 2005;43(11):1130-9.
11. University of Manitoba. Concept: Charlson Comorbidity Index 2021 [Available from: http://mchp-appserv.cpe.umanitoba.ca/viewConcept.php?conceptID=1098.
12. Quan H, Li B, Saunders LD, Parsons GA, Nilsson CI, Alibhai A, et al. Assessing validity of ICD-9-CM and ICD-10 administrative data in recording clinical conditions in a unique dually coded database. Health Serv Res. 2008;43(4):1424-41.
13. Clark DO, Von Korff M, Saunders K, Baluch WM, Simon GE. A chronic disease score with empirically derived weights. Med Care. 1995;33(8):783-95.
14. BC Centre for Disease Control. Chronic Disease Dashboard: Case Definitions 2020 [Available from: http://www.bccdc.ca/health-professionals/data-reports/chronic-disease-dashboard#Case--Definitions.
15. British Columbia Ministry of Health. British Columbia Chronic Disease Registries (BCCDR): Methods Overview 2020 [Available from: http://www.bccdc.ca/resource-gallery/Documents/Chronic-Disease-Dashboard/BC%20CDR%20Methodology%20Overview%202018-19.pdf.
16. Alaghehbandan R, Macdonald D, Barrett B, Collins K, Chen Y. Using administrative databases in the surveillance of depressive disorders--case definitions. Popul Health Manag. 2012;15(6):372-80.

© 2024 Min JE et al. *JAMA Network Open.*

17. Townsend L, Walkup JT, Crystal S, Olfson M. A systematic review of validated methods for identifying depression using administrative data. Pharmacoepidem Dr S. 2012;21:163-73.
18. Davis KA, Sudlow CL, Hotopf M. Can mental health diagnoses in administrative data be used for research? A systematic review of the accuracy of routinely collected diagnoses. BMC Psychiatry. 2016;16:263.
19. Oiesvold T, Nivison M, Hansen V, Sorgaard KW, Ostensen L, Skre I. Classification of bipolar disorder in psychiatric hospital. A prospective cohort study. BMC Psychiatry. 2012;12:13.
20. Sellgren C, Landen M, Lichtenstein P, Hultman CM, Langstrom N. Validity of bipolar disorder hospital discharge diagnoses: file review and multiple register linkage in Sweden. Acta Psychiatr Scand. 2011;124(6):447-53.
21. Kurdyak P, Lin E, Green D, Vigod S. Validation of a Population-Based Algorithm to Detect Chronic Psychotic Illness. Can J Psychiatry. 2015;60(8):362-8.
22. Goldner EM, Jones W, Waraich P. Using administrative data to analyze the prevalence and distribution of schizophrenic disorders. Psychiatr Serv. 2003;54(7):1017-21.
23. Nosyk B, Colley G, Yip B, Chan K, Heath K, Lima VD, et al. Application and validation of case-finding algorithms for identifying individuals with human immunodeficiency virus from administrative data in British Columbia, Canada. PloS one. 2013;8(1):e54416.
24. Shah H, Bilodeau M, Burak KW, Cooper C, Klein M, Ramji A, et al. The management of chronic hepatitis C: 2018 guideline update from the Canadian Association for the Study of the Liver. CMAJ. 2018;190(22):E677-E87.
25. Perrone V, Sangiorgi D, Buda S, Degli Esposti L. Disease progression and health care resource consumption in patients affected by hepatitis C virus in real practice setting. Clinicoecon Outcomes Res. 2016;8:591-7.
26. Kim D, Li AA, Gadiparthi C, Khan MA, Cholankeril G, Glenn JS, et al. Changing Trends in Etiology-Based Annual Mortality From Chronic Liver Disease, From 2007 Through 2016. Gastroenterology. 2018;155(4):1154-63.e3.
27. Sheu MJ, Chin TW, Ku FP, Li CY, Li ST, Lu TH. Validation of coding algorithms for identifying people with viral hepatitis using claims data according to different standard references. BMC Infect Dis. 2022;22(1):222.
28. Degenhardt L, Randall D, Hall W, Law M, Butler T, Burns L. Mortality among clients of a state-wide opioid pharmacotherapy program over 20 years: risk factors and lives saved. Drug Alcohol Depend. 2009;105(1-2):9–15.
29. Hagstrom H, Adams LA, Allen AM, Byrne CD, Chang Y, Gronbaek H, et al. Administrative Coding in Electronic Health Care Record-Based Research of NAFLD: An Expert Panel Consensus Statement. Hepatology. 2021;74(1):474-82.
30. Centers for Disease Control and Prevention. Alcohol-Related ICD Codes 2020 [Available from: https://www.cdc.gov/alcohol/ardi/alcohol-related-icd-codes.html.
31. Tian TY, Zlateva I, Anderson DR. Using electronic health records data to identify patients with chronic pain in a primary care setting. J Am Med Inform Assoc. 2013;20(e2):e275-80.
32. Tonelli M, Wiebe N, Fortin M, Guthrie B, Hemmelgarn BR, James MT, et al. Methods for identifying 30 chronic conditions: application to administrative data. BMC Med Inform Decis Mak. 2015;15:31.
33. Tonelli M, Wiebe N, Fortin M, Guthrie B, Hemmelgarn BR, James MT, et al. Correction to: Methods for identifying 30 chronic conditions: application to administrative data. BMC Med Inform Decis Mak. 2019;19(1):177.
34. Center of excellence for health systems improvement. ICD-10 Tobacco Billing Guide [Available from: https://tobaccofreeny.org/images/PDFs/ICD-10_Tobacco_Billing_Guide.pdf.

© 2024 Min JE et al. *JAMA Network Open.*

35. Government of British Columbia. Diagnostic Code Descriptions (ICD-9). [Available from: https://www2.gov.bc.ca/gov/content/health/practitioner-professional-resources/msp/physicians/diagnostic-code-descriptions-icd-9.
36. World Health Organization. International statistical classification of diseases and related health problems (10th ed.). 2019 [Available from: https://icd.who.int/browse10/2019/en.
37. Government of British Columbia. BC PharmaCare plans [Available from: https://www2.gov.bc.ca/gov/content/health/health-drug-coverage/pharmacare-for-bc-residents/who-we-cover#planp.
38. Schneeweiss S, Rassen JA, Glynn RJ, Avorn J, Mogun H, Brookhart MA. High-dimensional propensity score adjustment in studies of treatment effects using health care claims data. Epidemiology. 2009;20(4):512-22.
39. Rassen JA, Doherty M, Huang W, Schneeweiss S. Pharmacoepidemiology Toolbox. Boston, MA.2014 [Available from: https://www.drugepi.org/dope/software#Pharmacoepidemiology.
40. Rassen JA, Shelat AA, Myers J, Glynn RJ, Rothman KJ, Schneeweiss S. One-to-many propensity score matching in cohort studies. Pharmacoepidemiol Drug Saf. 2012;21 Suppl 2:69-80.
41. Austin P. Optimal Caliper Widths for Propensity-Score Matching When Estimating Differences in Means and Differences in Proportions in Observational Studies. Pharmaceutical statistics. 2011;10:150-61.

© 2024 Min JE et al. *JAMA Network Open.*
