# Supplementary material for: Opioid Coprescription Through Risk Mitigation Guidance and Opioid Agonist Treatment Receipt
Source: JAMA Netw Open. 2024 May 15;7(5):e2411389. doi: 10.1001/jamanetworkopen.2024.11389 (PMC11096992; doi:10.1001/jamanetworkopen.2024.11389)
Supplement: Supplement 2. — Data Sharing Statement [file jamanetwopen-e2411389-s002.pdf]

Data Sharing Statement

Min. Opioid Coprescription Through Risk Mitigation Guidance and Opioid Agonist Treatment Receipt. *JAMA Netw Open*. Published May 15, 2024.
doi:10.1001/jamanetworkopen.2024.11389

Data
Data available: No

Additional Information
Explanation for why data not available: The datasets generated and/or analyzed during the current study are not publicly available due to concerns on individual level administrative data and privacy restrictions. Materials and code available on request.
